# Inducible Bronchus-Associated Lymphoid Tissue: Taming Inflammation in the Lung

**DOI:** 10.3389/fimmu.2016.00258

**Published:** 2016-06-30

**Authors:** Ji Young Hwang, Troy D. Randall, Aaron Silva-Sanchez

**Affiliations:** ^1^Division of Clinical Immunology and Rheumatology, Department of Medicine, University of Alabama at Birmingham, Birmingham, AL, USA

**Keywords:** inducible bronchus-associated lymphoid tissue, tertiary lymphoid organ, ectopic lymphoid organ, lymphoid neogenesis, germinal center

## Abstract

Following pulmonary inflammation, leukocytes that infiltrate the lung often assemble into structures known as inducible Bronchus-Associated Lymphoid Tissue (iBALT). Like conventional lymphoid organs, areas of iBALT have segregated B and T cell areas, specialized stromal cells, high endothelial venules, and lymphatic vessels. After inflammation is resolved, iBALT is maintained for months, independently of inflammation. Once iBALT is formed, it participates in immune responses to pulmonary antigens, including those that are unrelated to the iBALT-initiating antigen, and often alters the clinical course of disease. However, the mechanisms that govern immune responses in iBALT and determine how iBALT impacts local and systemic immunity are poorly understood. Here, we review our current understanding of iBALT formation and discuss how iBALT participates in pulmonary immunity.

## Introduction

The evolutionary emergence of lymphocytes with diversified antigen receptors allows the immune system to recognize and respond to a myriad of unknown antigens. However, despite the enormous number of B cells and T cells in the naive compartment, the frequency of B cells or T cells with any particular specificity is miniscule, necessitating efficient mechanisms to acquire and present antigens to the responding lymphocytes (1). Moreover, B and T cells of the same specificity must find one another and interact in a cognate way in order to differentiate into effector cells (2). In order to accomplish these goals, the immune system has evolved a system of secondary lymphoid organs (3).

Secondary lymphoid organs, such as spleen, lymph nodes, Peyer’s patches, and other mucosa-associated lymphoid tissues, recruit naive B and T cells from the blood and sample antigens from local non-lymphoid organs and mucosal surfaces, thereby allowing naive lymphocytes to efficiently peruse antigens from all the tissues of an entire organism without having to migrate through those tissues themselves (4). Moreover, secondary lymphoid organs are highly organized and contain architectural domains that facilitate sequential cellular interactions between antigen-presenting cells and lymphocytes and efficiently promote B and T cell activation, selection, and differentiation (1) – ultimately increasing the efficiency of the immune response.

Mammals, birds, and bony fish have easily recognizable secondary lymphoid organs and tissues with some of the characteristics of secondary lymphoid organs are observed in the gut lamina propria of cartilaginous fish, such as sharks (5). In fact, the appearance of cell clusters containing two types of adaptive immune cells can be traced back to pharynx of the lamprey (6), a jawless vertebrate and one of the oldest organisms to have an adaptive immune system (7). Thus, most vertebrates have evolved some type of tissue that is specialized to promote interactions between various cells of the adaptive immune system. Other authors have recently reviewed the evolutionary aspects of lymphoid organs (5); therefore, in this review, we will focus only on the developmental and functional aspects of lymphoid tissues in the lung.

Most secondary lymphoid organs in mice and humans develop embryonically in the absence of microbial stimulation or foreign antigens (8). However, the structure and function of some secondary lymphoid organs, particularly those at mucosal surfaces, is dramatically altered upon exposure to environmental antigens and commensal organisms (9). For example, Peyer’s patches in the small intestine dramatically increase in size and complexity following commensal colonization (10, 11). Similarly, Nasal-Associated Lymphoid Tissue in rodents does not completely develop until after birth and this process is accelerated by microbial exposure (12). Strikingly, the appendix of rabbits is both a primary and secondary lymphoid tissue that is functionally dependent on microbial colonization (13). More importantly, however, some lymphoid tissues, known as tertiary lymphoid tissues, develop *only* after environmental exposure to microbes, pathogens, or inflammatory stimuli. Tertiary lymphoid tissues form in a wide variety of organs, including pancreas (14), thyroid (15), thymus (16), salivary gland (17, 18), brain (19), liver (20), kidney (21), and others (22), but in this review, we will focus on tertiary lymphoid tissue that forms in the lung, known as inducible Bronchus-Associated Lymphoid Tissue or iBALT.

Although the lungs of mice and humans normally lack organized lymphoid tissue, areas of iBALT form in the lungs following some types of infection or inflammation (23, 24) (Table 1). iBALT is a classic example of a tertiary lymphoid tissue, since it does not develop in a pre-programed way and its occurrence, size, and number in the lung depends on the type and duration of antigenic exposure (25, 26). Areas of iBALT are observed in the lungs of mammals (27–31) and birds (32–34) and are likely found in all air-breathing vertebrates. However, iBALT is most well characterized in the lungs of rodents and humans. Here we will summarize below the results of studies from these species.

**Table 1:** **Association of iBALT with infectious and inflammatory diseases of the lung**.

Disease	Important finding	Reference
COPD	SERPINEE2 prevents iBALT formation, inhibits thrombin	(119)
	CXCL13 expression associated with iBALT	(190)
	iBALT associated with COPD stage	(192)
	iBALT associated with uptake of pulmonary antigens	(198)
	Increase in dendritic cells in iBALT of COPD patients	(199)
	CCL20-driven accumulation of dendritic cells in iBALT	(200)
	Increased B follicles in COPD patients	(201)
	iBALT found in smokers and asthmatics	(210)
	CCR7 involved in iBALT formation after cigarette smoke	(195)
Particulate exposure	Exposure to diesel exhaust particles promotes iBALT	(88)
	Cigarette smoke-induced iBALT	(89)
	iBALT associated with response to silica	(226)
Pulmonary arterial hypertension	Formation of iBALT in patients with PAH	(92)
	Association of IL-17 in the formation of iBALT in PAH	(93)
Hypersensitivity pneumonitis	iBALT associated with hypersensitivity pneumonitis	(189)
	iBALT areas found in hypersensitivity pneumonitis	(209)
Rheumatoid lung disease	iBALT found in patients with rheumatoid lung disease	(208)
Sjogren syndrome	IL-22 promotes CXCL13 expression and iBALT formation	(18)
Allograft rejection	iBALT formation associated with lung transplant rejection	(218)
	iBALT formation associated with lung transplant tolerance	(222)
Allergy/asthma	Pulmonary challenge of rats with antigens	(35)
	Pulmonary challenge of rats with HRP	(62)
	Pulmonary challenge with OVA leads to IgE in iBALT	(90)
	IL-5 overexpression and eosinophils lead to iBALT	(129)
	iBALT is sufficient for immunity to allergens	(176)
	Local IgE production in iBALT in aspergillosis	(211)
	Poor association of iBALT with asthma in non-smokers	(212)
Viral infection	iBALT independently promotes immunity to influenza	(23)
	iBALT in mink infected with Aleutian disease virus	(28)
	CXCL13, CCL19, and CCL21 are important for iBALT function	(56)
	iBALT formation after infection with modified vaccinia ankara	(65)
	iBALT accelerates immunity to pneumovirus	(82)
	Infection of mice with murine cytomegalovirus	(85)
	Dendritic cell – dependence of iBALT	(121)
	Immunologic memory maintained in iBALT	(180)
	iBALT-mediated immunity to SARS, influenza	(181)
	Acceleration of CD4 responses by iBALT	(67)
Bacterial infection	iBALT in humans with bacterial infections	(29)
	iBALT in goats with *Pasteurella haemolytica*	(31)
	Lymphatics around iBALT after *Mycoplasma pulmonis* infection	(64)
	Infection of pigs with *Actinobacillus pleuropneumoniae*	(25)
	Pulmonary exposure to LPS leads to IL-17-dependent iBALT	(78)
	iBALT in pigs exposed to hemolytic *streptococcus*	(81)
	Formation of iBALT in human fetuses with amnionitis	(83)
	*Mycobacterium tuberculosis* induces iBALT in mice	(87)
	IL-17-dependent iBALT formation *Pseudomonas aruginosa*	(94)
	IL-17-dependent CXCL13 after *M. tuberculosis*	(100)
	IL-23 maintains iBALT and granulomas in *M tuberculosis*	(98)
	iBALT is sufficient for immunity to *M tuberculosis*	(175)
	iBALT is sufficient for immunity to *M tuberculosis*	(177)
	Pulmonary vaccination to *F. tulerensis* promotes iBALT	(182)
	Lymphoid chemokines maintain iBALT in *tuberculosis*	(185)
	iBALT recruits CXCR5 + T cells in *tuberculosis*	(186)
	Human tuberculosis granulomas resemble iBALT	(187)
	Vaccination elicits iBALT and protects from tuberculosis	(188)
Lung cancer	iBALT associated with good prognosis in lung cancer	(224)
	iBALT associated with ILC3 cells in lung cancer	(225)
Spontaneous iBALT	IL-6 overexpression leads to iBALT	(130)
	Oncostatin M overexpression leads to iBALT	(131)
	Poor Treg function in CCR7^−/−^ mice leads to iBALT	(127)
Lung fibrosis	Reduced bleomycin-induced fibrosis in lungs with iBALT	(227)
	Reduced bleomycin-induced fibrosis in lungs with iBALT	(228)

## General Features of iBALT

**Figure 1:**
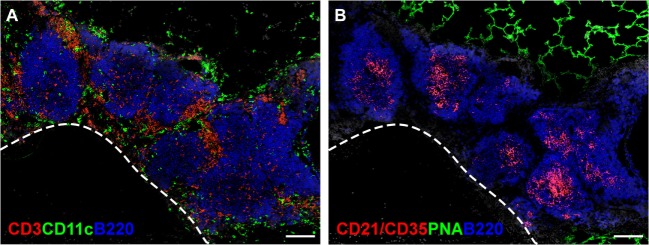
**The structure of iBALT**. C57BL/6 mice were intranasally administered LPS on days 3, 5, 7, 9, and 11 after birth, and lungs were obtained 6 weeks after the last LPS administration. **(A)** Frozen sections were probed with anti-CD3 (red), anti-CD11c (green), and anti-B220 (Blue), and images were acquired on a Nikon Eclipse Ti microscope using the 20× objective. The dashed line indicates the position of a blood vessel. Scale bar indicates 200 μm. **(B)** Frozen sections were probed with anti-CD21/35 (red), peanut agglutinin (PNA - green), and anti-B220 (blue), and images were acquired on a Nikon Eclipse Ti microscope using the 20× objective. Scale bar indicates 200 μm.

As the name indicates, iBALT does not occur at random sites in the lungs, but develops in close proximity to the basal side of the bronchial epithelium (35), often in the perivascular space of pulmonary blood vessels (36, 37). The leukocytes comprising iBALT are arranged in two zones, the B cell follicle and the T cell zone (37), in a way that resembles the organization of conventional secondary lymphoid organs. The B cell follicles of iBALT contain tight clusters of IgD^+^ follicular B cells grouped around a network of stromal cells, known as follicular dendritic cells (FDCs), that express CD21, CXCL13, and lymphotoxin (LT) β receptor (LTβR) (38–41) (Figure 1B). B cell follicles in reactive iBALT areas may contain large germinal centers (23), in which B cells are rapidly dividing in response to antigen. These germinal centers will also contain activated CD4 T cells, known as T follicular helper (Tfh) cells (42, 43) (Figure 1A). The T cell zone of iBALT surrounds the B cell follicles and contains CD4 and CD8 T cells as well as conventional dendritic cells (DCs) (24, 44) (Figure 1A).

The compartmentalization of B and T cell areas in iBALT requires specialized fibroblastic cells, usually referred to as stromal cells. Stromal cells in the B cell follicle are primarily FDCs, which express CXCL13, a chemokine that attracts CXCL13-expressing cells like B cells and Tfh cells (42, 45, 46). Stromal cells are also observed in the T cell zones of iBALT and are likely similar to the fibroblastic reticular cells (FRCs) found in the T zones of conventional secondary lymphoid organs (47, 48). These cells express chemokines like CCL19 and CCL21 (49–51), which attract naive T cells and activated DCs (45, 52, 53). T zone stromal cells also produce IL-7 (54, 55), a cytokine important for the survival of naive lymphocytes.

In addition to the stromal cells that support the B and T cell areas, iBALT often features high endothelial venules (HEVs) (56), which are specialized blood vessels that express homing and adhesion molecules as well as chemokines that together recruit lymphocytes from the blood (57). HEVs in iBALT are located just outside the B cell follicle in the T cell zone (56). Although one might assume that iBALT is a mucosal lymphoid tissue based on its location in the lung, the HEVs of iBALT express peripheral lymph node addressin (PNAd) like the HEVs of peripheral lymph nodes (56), but do not express mucosal addressin cell adhesion molecule (MAdCAM), which is prominently expressed by mesenteric lymph nodes and Peyer’s patches in the intestine (58). HEVs in iBALT also express (or display) CCL21 (56), which is likely important for the recruitment of naive lymphocytes from the blood.

Given that iBALT is located underneath the bronchial epithelium, one might assume that it acquires antigens directly from the lumen of the airways *via* epithelial M cells. Although antigen-transporting M cells have been reported in the iBALT of some species (35, 59, 60), they are not consistently observed and many areas of iBALT do not have the classic structure of a mucosal lymphoid tissue, with a dome epithelium overlaying the B cell follicle (61). It is not clear at this time whether this inconsistency in the structure of iBALT is due to differences in species, the way in which iBALT is formed or the duration/magnitude of antigen exposure (62).

Despite the lack of obvious M cells in many iBALT areas, there are also lymphatic vessels that surround the B cell follicle and likely facilitate the uptake of antigens. In the normal lung, lymphatics originate from two distinct locations, one set of lymphatics originate from the parenchyma and follows the pulmonary veins toward the draining lymph node, and the other set originates around the connective tissue between the airways and veins, and follows the airways toward the draining lymph node (63). New data show that additional lymphatic vessels are generated during lung inflammation surrounding the iBALT areas, apparently by sprouting from the existing lymphatic network (64). Given the placement of iBALT in the perivascular space next to large airways, we expect that afferent lymphatics drain from the distal portions of the lung toward iBALT. The best evidence for this model is the ability of iBALT to collect labeled DCs and particulates (23, 64, 65).

Lymph nodes have both afferent and efferent lymphatics, whereas most mucosal lymphoid tissues have only efferent lymphatics that connect to downstream lymph nodes and ultimately to the blood. We assume that many of the lymphatics associated with iBALT are efferent lymphatics that allow cells within iBALT to re-enter the circulation. In fact, we expect that iBALT follows the conventional model of lymphoid recirculation in which naive B and T cells enter iBALT from the blood through PNAd-expressing HEVs and then exit iBALT *via* efferent lymphatic vessels (66). Efferent lymphatic vessels would also allow activated effector and memory lymphocytes to exit iBALT and re-enter the circulation. Although the ability of efferent lymphatics to collect cells primed in iBALT and drain them to the downstream mediastinal lymph node has not been directly demonstrated, recent data show that the presence of iBALT promotes more rapid responses in the draining LN (67), suggesting that iBALT is connected to downstream lymph nodes and can alter the trafficking of antigen-bearing DCs and primed lymphocytes. Importantly, new data show that lymphatic endothelial cells in iBALT areas are more than just highways for leukocyte trafficking. In addition to producing the chemoattractant, CCL21, lymphatic endothelial cells also produce IL-7 and contribute to the maintenance of memory T cells (68). Thus, the lymphatic vessels surrounding iBALT likely have multiple functions.

## iBALT Development

Secondary lymphoid organs, such as lymph nodes and Peyer’s patches, form independently of antigenic or inflammatory stimuli in a highly ordered process that occurs during embryogenesis at very specific times (8) and reviewed in Ref. (69). Once that developmental window is passed, lymph nodes are no longer able to develop, even if all the necessary cells and molecules are present (8). In contrast, the development of iBALT requires an inflammatory or infectious stimulus in most species (25, 26, 70–73), including rats (74–77), mice (78), goats (79), chicken (33), and humans (29, 73, 80), and its development can be initiated throughout life. In contrast, pigs are reported to form iBALT in the lungs during fetal development (81). However, it is unclear whether this observation reflects a species or developmental difference.

Although the formation of iBALT is not restricted to a developmental window during embryogenesis, it seems to form more easily in the neonatal period just after birth (78, 82). For example, iBALT is found in the lungs of healthy adult humans at a relatively low frequency (83) but is found with increasing frequencies in the lungs of children and infants (29, 73, 83). The incidence of iBALT increases dramatically in all age groups following infection (70, 72, 73) but is highest in the lungs of infected children and infants and, most strikingly, is a prominent feature in 100% of late-term fetuses miscarried as a result of amnionitis (29), which results from an *in utero* pulmonary infection.

In part, the increased frequency of iBALT in the lungs of neonates and infants might reflect the initial exposure of a naive individual to stimuli such as pulmonary pathogens, microbial products, and allergens (25). However, the neonatal immune system also seems to favor the development of iBALT and other tertiary lymphoid tissues in mice living in controlled environments (78, 82, 84). For example, the injection of cell suspensions from dissociated lymph nodes into the skin of neonatal mice leads to the formation of highly organized lymphoid tissues (84), whereas the injection of the same cells into adults does not (84). Similarly, the repeated intranasal administration of the microbial product, LPS, to neonatal C57BL/6 mice induces iBALT formation, whereas repeated intranasal administration of LPS to weanling or adult C57BL/6 mice does not (78, 82). In another example, pulmonary infection of neonatal mice with cytomegalovirus (CMV) promotes the formation of Nodular Inflammatory Foci (NIF), whereas the pulmonary infection of adult mice with CMV does not (85, 86). NIFs are similar to iBALT in that they seem to support adaptive immune responses in the lung, but NIFs lack a B cell follicle and contain mostly a mix of CD8 T cells and DCs (85). At this point, it is unclear whether BALT and NIF formation are products of two different types of immune responses or whether CMV diverts the immune response leading to NIF formation as a byproduct and preventing BALT formation.

Interestingly, the preferential ability of neonates to form tertiary lymphoid tissues is less striking in BALB/c mice, as the pulmonary administration of LPS on a single day is sufficient to trigger iBALT formation in both neonatal and adult BALB/c mice (82). Moreover, other investigators have observed iBALT formation in adult mice following a variety of pulmonary challenges, including infections (87), particulates (88, 89), and allergens (90). Thus, the ability to trigger iBALT formation (or NIF formation) at particular stages of development likely reflects the inflammatory environment at the time of challenge and the type and duration of the challenge.

**Figure 2:**
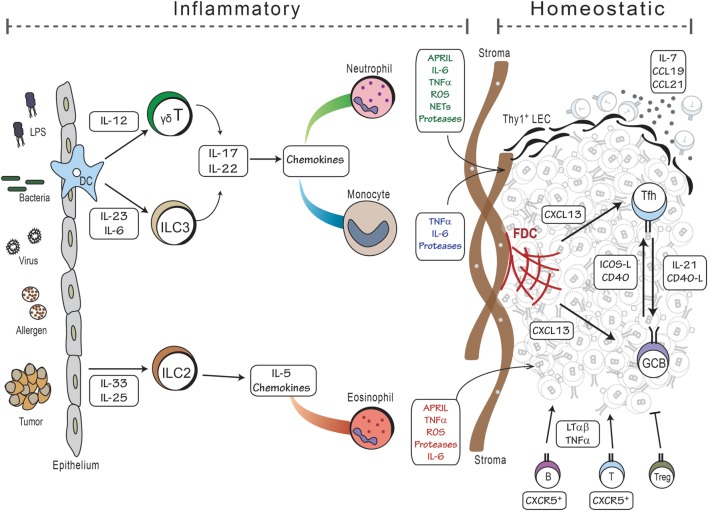
**Model of iBALT development**. The development of iBALT can be initiated by a wide variety of stimuli, including microbial products, bacteria, viruses, allergens, tumors, and particulates (left side), which trigger the activation and cytokine production from epithelial cells and dendritic cells. Innate cells, such as ILCs and γδT cells, become activated and produce cytokines and chemokines that attract inflammatory cells like neutrophils, monocytes, and eosinophils. Granulocytes produce cytokines that promote B cell activation as well as proteases and reactive oxygen that activate stromal cell precursors. These activities would all occur during an inflammatory process. Once mature B and T cells are recruited to the lung, they reinforce the differentiation of stromal cells into mature FDCs and FRCs that respectively support the B and T cell areas of iBALT. Once inflammation is resolved, the lymphocytes, dendritic cells, and stromal cells can maintain the iBALT structure using homeostatic mechanisms – lymphotoxin and chemokines – for months.

Given that the structure of iBALT is similar to that of conventional secondary lymphoid organs, it is not too surprising that the cytokines and chemokines (as well as their receptors) that are important for the development of secondary lymphoid organs are also important for the development of iBALT. For example, CXCL13 and its receptor, CXCR5 are required for the formation and maintenance of B cell follicles in both secondary lymphoid organs (45) and in iBALT (56). Similarly, the ligands for CCR7, CCL19, and CCL21 are important for the organization of the T cell zone and for the recruitment of lymphocytes from the blood through HEVs in both conventional lymphoid organs (45) and iBALT (56). Moreover, under steady state conditions, the expression of CXCL13, CCL19, and CCL21 is controlled by LT signaling through its receptors, LTβR and TNFR1 in both lymph nodes (91) and iBALT (78). However, during iBALT development, the expression of CXCL13 and CCL19 is controlled by IL-17 and possibly other inflammatory cytokines – independently of LT (78). Although IL-17 promotes the expression of CXCL13, CCL19, and other inflammatory chemokines during iBALT development, once iBALT is formed and inflammation is resolved, the expression of CXCL13 and CCL19 is maintained by LT signaling, independently of IL-17 (78). Thus, LT and IL-17 act at different times during iBALT development (Figure 2).

IL-17 is also important for iBALT formation in patients with pulmonary arterial hypertension (92, 93). As might be expected, the expression of lymphoid chemokines, CXCL13, CCL19, and CCL21 in the iBALT areas of these patients correlated with the frequency of RORγt-expressing T cells, presumably Th17 cells (92). Similarly, the pulmonary administration of heat-killed *Pseudomonas aeruginosa* (HK-Pa) to mice promotes iBALT formation in an IL-17-dependent fashion (94). Interestingly, in HK-Pa-treated mice, IL-17 mediates the aggregation of B cells by CXCL12, rather than CXCL13 (94). However, IL-17 is not always required for the formation of iBALT. For example, pulmonary infection of IL-17-deficient mice with Modified Vaccinia virus Ankara (MVA) promotes the formation of a classic iBALT structure (94). Despite the absence of IL-17, lymphoid chemokines, such as CXCL13 are still expressed, possibly as a result of reduced Treg activity (95) or complement-mediated neutrophil recruitment (96) as will be discussed later in this review. Thus, there are multiple ways to recruit lymphocytes to the lung and organize them into iBALT-type structures. Nevertheless, IL-17 seems to be an important cytokine in the development of ectopic lymphoid tissues in multiple locations (19, 97).

IL-22 is also important for iBALT formation (18, 98). Although IL-22 is most well known to act on epithelial cells in the lung and gut to trigger anti-microbial defenses and promote epithelial repair (99), the IL-22 receptor is also expressed on stromal cells in the B cell follicle of iBALT (18). In addition, the LPS-induced development of iBALT is impaired in the absence of IL-22 (18). Moreover, the lymphoid domains of tuberculosis granulomas, which resemble iBALT, are also disrupted in the absence of IL-22 – the FDC network is smaller, the B cell follicle is smaller and CXCL13 expression is reduced (98, 100). IL-22 is also important for the formation of other ectopic follicles, as the overexpression of IL-22 in the salivary gland strongly promotes the formation of ectopic follicles in that tissue (18). Interestingly, the IL-22 receptor is also expressed by stromal cells and FDCs in other locations, particularly following inflammation (101). However, IL-22 is not required for lymph node development or for the differentiation of stromal cells in lymph nodes (69). Thus, the requirement for IL-22 also distinguishes the development of iBALT from the development of conventional lymphoid tissues.

Another important difference in the development of lymph nodes and iBALT is the requirement for lymphoid tissue inducer (LTi) cells. LTi cells are a subset of innate lymphoid cells (ILCs) that are dependent on the transcription factors, RORγt and Id2, and express cytokines like TNF, LT, IL-22, and IL-17 (102, 103). LTi cells express CXCR5 and CCR7 and, during embryogenesis, home to developing lymph nodes (104), where they express LTα and LTβ and, through the actions of the LTβR and TNFR1, promote the differentiation of local mesenchymal cells into mature fibroblastic stromal cells that form the scaffold of secondary lymphoid organs (38, 105–109). Importantly, LTi cells are essential for the development of lymph nodes, as mice lacking RORγt or Id2 completely lack lymph nodes and Peyer’s patches (102, 110, 111). Given that LTi cells express IL-17, IL-22, and LT, one might expect that these cells would also be required for iBALT formation. However, mice lacking RORγt and Id2 (and therefore lacking LTi cells) generate fully formed iBALT structures in the lungs (78). Thus, LTi cells are not required (although they may be involved) in the development of iBALT (Figure 2).

The differential requirement for LTi cells in the development of iBALT and lymph nodes probably reflects the difference in when these tissues are formed. Conventional lymph nodes form during embryogenesis in the absence of antigen or inflammation in an environment that lacks mature B and T cells (8). In contrast, iBALT forms after birth following exposure to antigenic and inflammatory stimuli that trigger the activation of mature lymphocytes. Given that the neonatal lung has mature T cells, such as γδT cells and Tfh cells that express TNF, IL-17, IL-22, and LT (78); these cells may functionally replace LTi cells for promoting iBALT development in postnatal mice.

Although IL-17 promotes the expression of CXCL13 and CXCL12, which in turn recruit B cells and Tfh cells, this process may not entirely explain the role of IL-17 in iBALT formation. For example, IL-17 is most well known for promoting the expression of CXCL9, CXCL10, and CXCL11 (112, 113) as well as cytokines, like G-CSF, that strongly attract inflammatory cells like neutrophils. These chemokines and cytokines are also dramatically upregulated in an IL-17-dependent manner following pulmonary LPS exposure in neonates (78). In fact, large numbers of neutrophils are recruited to the lungs of LPS-exposed neonates and are required for iBALT formation (82). Neutrophils are important for the production of APRIL and IL-21, which maintain B cell activation and survival and thereby help to promote iBALT formation (82). Interestingly, neonates are prone to granulocytosis (114, 115), which may help to explain why the formation of iBALT occurs more easily in this age group.

Other studies also support the role of neurophils in the formatin of iBALT. For example, mice doubly deficient for the genes encoding the NQO1 and NQO2 proteins, two neutrophil-expressed enzymes that limit ROS generation, spontaneously develop iBALT (116). NQO1^−/−^NQO2^−/−^mice have increased numbers of granulocytes in the peripheral blood and, in the lungs, have areas of iBALT with elevated numbers of neutrophils (116). However, it is not clear from these studies whether the elevated production of reactive oxygen directly promotes iBALT or whether the elevated numbers of neutrophils in the lung perform some other function that promotes iBALT formation.

Neutrophils may also play a role in the spontaneous formation of iBALT in Serpine2-deficient (SE2^−/−^) mice. Serpine2 is a protease inhibitor that inhibits proteases like thrombin, trypsin, urokinase plasminogen activator (uPA), and plasmin, but not elastase (117, 118). These mice spontaneously develop iBALT in their lungs as early as 8 weeks after birth (119). The formation of iBALT in SE2^−/−^ mice is associated with the excessive expression of both inflammatory (CXCL9, CXCL10, and CXCL11) and homeostatic (CXCL12, CXCL19, and CCL19) chemokines as well as heightened thrombin activity (119). Importantly, the pulmonary administration of thrombin to the lungs of WT mice promotes an NFkB-dependent increase in chemokine expression from epithelial cells. Thrombin also activates protease-activated receptors (PARs) on neutrophils and endothelial cells and promotes neutrophil chemotaxis (120), suggesting that neutrophils may play a role in this process. Given the role of proteases in COPD, a lung disease in which iBALT may contribute to pathology, it is likely that protease-mediated processes will be an important contributing pathway in iBALT formation.

CD11c-expressing DCs are also important for the formation and the maintenance of iBALT. For example, mice depleted of CD11c-expressing cells rapidly lose existing areas of iBALT (94, 121) and the depletion of CD11c-expressing cells following the pulmonary administration of LPS to neonates prevents iBALT formation (78). Conversely, the pulmonary administration of bone marrow-derived DCs (BMDCs) promotes the formation of iBALT structures (121). CD11c-expressing DCs may promote iBALT formation/maintenance directly by providing LT (121) or indirectly by supporting the activation of T cells and B cells. In addition, depending on how they are activated, DCs are potent sources of IL-23, which acts on ILC3 cells (122, 123), γδT cells (124), and even CD4 T cells (125) to promote their production of IL-17. One caveat to the interpretation of these studies is that alveolar macrophages also express CD11c and will be deleted in CD11c-DTR mice. Moreover, BMDCs are actually a mix of true DCs and macrophages (126). Thus, macrophages may play an important, although poorly understood, role in the development and maintenance of iBALT.

Regulatory T cells (Tregs) limit local immune responses and, not surprisingly, can restrain the formation of iBALT. For example, CCR7-deficient mice spontaneously form iBALT (127), in part, because CCR7-deficient Tregs are poorly recruited to the lymph node, which prevents them from inhibiting effector T cell responses. This process can be mimicked by the blockade of CD62L-dependent lymph node homing (127). However, the interpretation of these studies is complicated. Does poor T cell homing to the lymph node lead to increased homing to the lung, regardless of whether Tregs are working properly? Does depletion of Tregs or impairment of their activity promote autoimmunity and therefore local pulmonary inflammation and the development of iBALT? There is also a connection between Tregs and neutrophils, as the selective depletion of FoxP3^+^ Tregs in neonatal mice increases the number of neutrophils and promotes iBALT formation (82), whereas the targeted depletion of neutrophils significantly reduces the number and size of iBALT areas following intranasal LPS administration. Thus, Tregs play an inhibitory role in iBALT development consistent with their immunosuppressive activities.

Although the mechanisms that recruit leukocytes to the lung are clearly important in the formation of iBALT, the resolution of pulmonary inflammation will also likely play a role. In other words, if leukocytes are recruited to a site faster than they can be cleared, then they will build up over time and, upon reaching a critical mass, may spontaneously assemble into a lymphoid tissue like iBALT (128). In support of this idea, the treatment of mice with the S1P1R agonist, FTY720, also promotes iBALT development, possibly by retaining cells in the lungs (127). One mechanism for clearing cells from the lung is drainage *via* lymphatic vessels (64), which are concentrated surrounding iBALT areas (64). In fact, infection of mice with *Mycoplasma pulmonis* induces large areas of iBALT and increases the number and volume of intrapulmonary lymphatics (64). The increase in lymphatics is mediated by signaling through VEGF-R2 and VEGF-R3. However, the simultaneous blockade of both receptors does not impede iBALT development (64). These data suggest that differentiation of lung lymphatics and VEGF play a marginal role in the development of iBALT; however, the newly generated lymphatics surrounding iBALT areas are likely to be important in regulating pulmonary inflammation and edema in response to subsequent respiratory infections.

Although the development of iBALT following exposure to microbes or microbial products provides information about normal physiological processes, these types of experiments are complicated to interpret due to the wide array of pathways that may be triggered by infection. To avoid this problem, some investigators have used the reductionist approach of overexpressing individual cytokines in the lung. In three separate reports, all of them using an adenovirus expression system, the forced overexpression of IL-5 (129), human IL-6/IL16R (130), or the IL-6 family member, oncostatin M (OSM) (131) in mouse lungs successfully generated iBALT structures. Interestingly, these pro-inflammatory cytokines have the potential to activate B cells (129, 131–133), which we know are important for the production of LT and the differentiation of lung stromal cells into FDCs. However, the overexpression of OSM and IL-5 also promoted the accumulation of eosinophils in the lungs. Thus, the local activation of eosinophils may functionally replace the role of neutrophils in these circumstances and provide cytokines, reactive oxygen, or proteases that facilitate the formation of iBALT.

## Toward a Model of iBALT Development

The formation of iBALT depends on pulmonary infection or inflammation, and it seems that a wide variety of stimuli, including bacteria, viruses, microbial products, allergens, and even tumors, are capable of triggering this process (Figure 2). In most cases, repetitive exposures (LPS, allergens), infectious agents (viruses, bacteria), or long-lasting stimuli (particulates) are required, suggesting that a transient inflammatory response is generally not sufficient to promote iBALT formation. Given the diverse nature of the stimuli capable of inducing iBALT formation; it is difficult to find a single pathway that is common to all. However, the recruitment of granulocytes (neutrophils or eosinophils) does seem to be a prominent feature of most models. Importantly, both neutrophils and eosinophils produce a variety of cytokines that help FDC differentiation (TNFα, LTβ), B cell activation (APRIL, IL-6), and promote the recruitment of more neutrophils (IL-23, G-CSF) or eosinophils (eotaxin, IL-5) (134, 135). They also make proteases and reactive oxygen that likely trigger receptors or cause damage in a way that promotes the accumulation of activated lymphocytes (136–138). These processes seem to be particularly active in neonates, perhaps because neonates are prone to heightened neutrophilia (114, 139), have a relatively high frequency of IL-17-producing γδ-T cells (140) or ILCs (103), and a relatively low frequency of Tregs (141–143).

Additional neutrophil functions may also be important for iBALT formation or function. For example, during acute inflammation, neutrophils die *via* Fas-mediated apoptosis (144), and are subsequently cleared by macrophages (145). However, neutrophils may also die in a way that leads to the production of neutrophil extracellular traps (NETs) – a process called NETosis (146). Exposure of neutrophils to reactive-oxygen species as well as activation by LPS, IFNγ, or CXCL8 can favor NETosis over apoptosis (147), and lead to an increase in IL-23 and IL-17 (148), which would favor iBALT formation. A hallmark characteristic of NETs is that neutrophil granule contents (histones, antimicrobial peptides, neutrophil elastase, and cytokines) remain attached to the expulsed DNA (147). Moreover, NET production is associated with lung fibroblast differentiation (149), as well as the processing and bioactivation of IL-33 by elastase (150), which triggers IL-17F production by bronchial epithelial cells (151). Once produced, IL-17 may become trapped on the NETs (149) and further increase the neutrophil recruitment to the lungs and the differentiation/activation of lung stroma, again leading to iBALT formation (Figure 2). This idea is consistent with data showing that NETs contribute to the control of pulmonary infection with *Streptococcus suis* in pigs and promote iBALT formation (152, 153).

Although iBALT development is triggered by inflammation, it can be maintained for months in the absence of inflammation by homeostatic mechanisms (78, 154). These mechanisms are the same as those that maintain the structure of conventional lymphoid tissues (Figure 1). For example, once B cell follicles are formed, B cells constitutively produce LT and TNF (41), which helps maintain the FDC network, HEVs, and lymphatic vessels (41). In turn, the stromal cells of the B and T cell zones make homeostatic cytokines, like IL-7 (54), and homeostatic chemokines, like CXCL12, CXCL13, CCL19, CCL20, and CCL21 (91), all of which act to recruit lymphocytes, direct their homing to the proper architectural domains, promote their survival and maintain the expression of LT and TNF, which support the stromal cells (91). Thus, once they are established, lymphocytes and stromal cells reinforce each other’s survival and differentiation in the absence of inflammatory cytokines or chemokines. Of course, in many chronic inflammatory conditions in the lung, both the inflammatory and homeostatic mechanisms operate simultaneously, which likely leads to continuous iBALT expansion and pathological outcomes.

Many of these same mechanisms are involved in the formation of tertiary lymphoid tissues in a variety of organs other than the lung. For example, tertiary lymphoid organs form in the brains of patients with multiple sclerosis (155, 156). The local expression of homeostatic chemokines, such as CXCL13, CCL19, and CCL21, correlates with the formation of these tissues (157) and soluble LTβR can suppress their formation and ameliorate the symptoms of EAE (156). Moreover, Th17 cells are involved in the pathogenesis of EAE and multiple sclerosis (158), and IL-17 is involved in lymphoid neogenesis by promoting the expression of lymphoid chemokines (19) and for the differentiation of local stromal cells (159). Thus, some of the same inflammatory and homeostatic pathways are involved in the formation of ectopic lymphoid tissues in the lungs and the brain.

Transgenic models also reveal similarities and differences between target organs in the formation of tertiary lymphoid tissues. For example, the pancreas develops tertiary lymphoid tissues, particularly in the context of diabetes (14, 160). CXCL13 is required for the organization of B cell follicles in the pancreas (161, 162), whereas chemokines like CCL21 and CCL19 are involved in recruiting B and T cells to the site and cytokines like IL-7 are important for their survival (49, 163, 164). Again, the LT and TNF signaling pathways are important for the maintenance of chemokine expression and the differentiation of stromal cells (164, 165), but their contribution to inflammation and diabetes is different (166). Interestingly, mice that express a CCL21 transgene in the pancreas develop well-defined ectopic lymphoid tissues, whereas mice expressing CCL21 in the skin do not (167). Thus, although there are clear commonalities in the pathways that promote ectopic lymphoid tissues in different organs, some striking differences that can probably be attributed to the different types of cells present in each target organ.

Exposure to a particular inflammatory stimulus will also likely dictate what pathways are involved in ectopic lymphoid tissue formation. For example, DCs (15, 168), CCL21 (169), and the LTβR (170) are required for the formation of ectopic follicles in the thyroid without a requirement for Id2-dependent LTi cells (15). However, the over-expression of CXCL13 in the gut promotes the formation of isolated lymphoid follicles *via* the recruitment of IL-22-expressing ILC3 cells (171). IL-17 is also involved in the formation of ectopic follicles in the gut (172), suggesting that Th17 responses to commensal organisms are likely driving the formation of tertiary lymphoid tissues in this location. Thus, the local inflammatory milieu and resident cell types likely dictate organ-specific pathways that promote the formation of tertiary lymphoid tissues in each non-lymphoid organ.

## Role of iBALT in Pulmonary Immune Responses to Infection

Given that iBALT structurally resembles conventional secondary lymphoid organs, one might assume that it performs similar functions, i.e., promoting encounters between naive lymphocytes that are recruited from the blood and antigen-presenting cells that have migrated from the lumen of the airways. However, this hypothesis is difficult to demonstrate experimentally. We and others have used LT-deficient mice, which lack conventional secondary lymphoid organs (173), to show that immune responses to a variety of antigens can be initiated directly in the lung (23, 174, 175). For example, LT-deficient mice generate nearly normal primary B and T cell responses to a pulmonary infection with influenza virus (23, 174). Similarly, LT-deficient mice are capable of generating primary immune responses following pulmonary exposure to allergens (176) and *Mycobacterium tuberculosis* (175, 177). Thus, conventional secondary lymphoid organs are not necessary for generating immune responses to the pulmonary antigens and pathogens that have been tested.

Despite their ability to generate primary immune responses, LT-deficient mice are not entirely immunocompetent. In particular, the DCs in LT-deficient mice have defects in survival and migration (178), in part due to poor expression of homeostatic chemokines. As a result, LT-deficient mice succumb to lower doses of influenza and fare worse than their normal counterparts, even though they do make primary immune responses. The generation of bone marrow chimeras (in which WT bone marrow is transferred into LT-deficient mice) circumvents the problems associated with LT deficiency, but does not restore lymph nodes or Peyer’s patches. Thus, upon the removal of the spleen, these mice lack all conventional secondary lymphoid organs and are known as Spleen, Lymph node and Peyer’s patch-deficient (SLP) mice. Importantly, SLP mice generate primary immune responses to influenza without any delay (23). Immune responses in SLP mice are initiated in the lung, in well-organized areas of iBALT. Moreover, germinal center responses are observed in the lungs of SLP mice and germinal enters can be observed in the B cell follicles of iBALT (69, 155, 179). In addition, influenza-specific memory T cells are generated and maintained in SLP mice, as are long-lived antibody-secreting cells (180). Most surprisingly, influenza-infected SLP mice fare better than WT mice, even in the absence of conventional secondary lymphoid organs (23). This result is likely due to slightly reduced T cell responses in SLP mice, which leads to reduced production of inflammatory cytokines, like TNF and IL-6, both of which contribute to weight loss and morbidity. Thus, iBALT areas in the lung are capable of generating primary immune responses, maintaining memory cells, and reducing morbidity and mortality associated with pulmonary infections.

Once iBALT is formed, it is maintained in the lungs for months, often in the absence of the original stimulus that triggered its formation (78). Moreover, once iBALT is formed, it acts like any other lymphoid organ and can recruit naive B and T cells and support their activation in response to antigens that are unrelated to the antigens that triggered iBALT formation. For instance, iBALT generated in response to *Mycobacterium tuberculosis* infection can recruit naive OVA-specific CD4 T cells and support their activation upon subsequent pulmonary exposure to OVA, without contributions from conventional secondary lymphoid organs (175, 177). Moreover, the transfer of DCs loaded with OVA peptide to mice in which iBALT was induced following pulmonary infection with MVA primes naive OVA-specific CD8 T cells in the lung (23, 65). Together, these data suggest that the specificity of naive T cell priming can be different from the antigenic stimulation that initially induced iBALT.

The presence of iBALT in normal mice also has dramatic consequences on the resulting immune response and clinical outcomes. For example, mice that have iBALT induced by pulmonary instillation of protein nanoparticles clear virus more rapidly and lose less weight following influenza infection (67, 181). In these mice, the kinetics of influenza-specific CD4^+^ T cells in the lymph node parallels that in the lung, suggesting that they are being primed in both locations simultaneously (67). The presence of iBALT also provides a beneficial effect with SARS-coronavirus and pneumovirus, which are cleared more rapidly in mice with iBALT by an accelerated antibody response (181). Similarly, mice that have iBALT induced as a result of neonatal LPS exposure lose less weight and clear pneumovirus faster than mice without iBALT (82). Importantly, the CD4 T cell response to pneumovirus is accelerated in mice with iBALT (82) suggesting that the presence of iBALT in the lung leads to faster, more efficient pulmonary immune responses that promote rapid viral clearance and reduce morbidity after infection. Thus, iBALT is beneficial in the context of respiratory virus infection.

The presence of iBALT is also protective in the context of bacterial infections. For example, intranasal vaccination with LPS and recombinant porin B from *Francisella tulerensis* induces highly organized iBALT structures (182) and confers improved survival and more efficient bacterial control upon challenge with the *Francisella tulerensis* vaccine strain (182). Similarly, iBALT induced with nanoparticles confers protection against subsequent challenge with *Coxiella burnetii* (181).

In addition to its role in resolving acute bacterial infections in the lung, iBALT also helps control chronic pulmonary infection with *Mycobacteria tuberculosis* (MTB). A hallmark of MTB infection is the formation of granulomas, clusters of lymphocytes that surround MTB-infected macrophages and contain infection (183). Granulomas exhibit many of the features of iBALT and B cell follicles containing germinal centers, and FDCs are often observed in MTB granulomas in mice (87, 184–186), humans (187), and monkeys (186, 188). Monkeys with latent MTB infection maintain large, well-organized areas of iBALT surrounding granulomas (186), whereas monkeys with active disease have fewer and less organized areas of iBALT. Thus, the maintenance of good iBALT structures seems to be important for the control of MTB. In fact, the activity of iBALT is sufficient to prime MTB-specific IFNγ-producing CD4 T cells and control infection, without contributions from conventional secondary lymphoid organs (175).

In fact, chronic pulmonary infection with MTB progressively leads to iBALT development, with progressive increases in the expression of both CCL19 and CXCL13 (87, 185). Importantly, the loss of these chemokines in CXCL13^−/−^ mice or *plt/plt* mice (lacking both CCL19 and CCL21) leads to disrupted iBALT architecture and delayed granuloma formation (185). CXCL13 seems to be most important for generating proper granulomas and for recruiting CXCR5-expressing T cells to the lungs (185), whereas granuloma formation is relatively normal in *plt/plt* mice, but the Th1 response is delayed (185). Consistent with the poor immune response in these mice, the titers of MTB are higher in the lungs of both CXCL13^−/−^ mice and in *plt/plt* mice and are even higher in the lungs of CXCL13^−/−^ x *plt/plt* mice.

Given that CXCL13 expression depends on IL-17 during pulmonary inflammatory conditions, it is not surprising that IL-17 is important in immunity to MTB following vaccination (184). In fact, intranasal vaccination of mice with MTB in combination with type II heat labile enterotoxin, elicits MTB-specific Th17 cells (100). Upon subsequent challenge with MTB, the memory Th17 cells elicit pulmonary expression of CXCL13, which recruits CXCR5-expressing T cells to the granuloma (100, 186). Consequently, MTB-specific Th1 cells activate macrophages and control infection.

Although iBALT seems consistently beneficial in the context of experimental models of infection, children and young adults with chronic or recurrent pneumonia develop iBALT areas in their lungs that are associated with bronchiolar damage (189), indicating that iBALT may contribute to bronchial pathology. Moreover, it is described in rabbits that collagens in the deeper layers of the bronchial wall are disrupted when iBALT is present (75), again supporting an association in pathologic context. However, the actual function of iBALT in these cases is difficult to assess.

## iBALT in Chronic Pulmonary Diseases

Patients with chronic pulmonary diseases often develop areas of iBALT. For example, patients with Chronic Obstructive Pulmonary Disease (COPD) develop areas of iBALT adjacent to their small airways (190). In fact, the Global Initiative for Chronic Obstructive Lung Disease (GOLD) has classified COPD patients into five-stages based on airflow limitation (191), and there is a strong correlation between the patients in GOLD stages 3 and 4 (severe and very severe) with the percentage of iBALT follicles in the airway and lymphocyte infiltration, compared to GOLD stages 1 and 2 (mild and moderate) patients (192). These studies also show a positive correlation between CXCL13 expression and lymphoid follicle density (192), suggesting that B cells accumulate in iBALT-like areas *via* a CXCL13-dependent mechanism. Interestingly, B cells in the iBALT areas of COPD patients may be capable of making their own CXCL13 (190). However, inflammatory chemokine receptors, such as CXCR3, are also found on B cells of COPD patients (193), suggesting a role of both inflammatory and homeostatic recruitment of B cells. As suggested in other studies, LT is also important for the formation of iBALT in the context of COPD (194, 195). Cytokines like BAFF are also important for the activation or survival of B cells in COPD lungs (196). B cells not only make antibodies but also activate macrophages in the context of COPD (197), which contributes to disease pathology. Similarly, Langerin-expressing DCs are found surrounding iBALT areas in COPD patients (198, 199) and their numbers positively correlate with the severity of COPD (199, 200). Consistent with these observations, mouse models of chronic cigarette smoke-induced COPD also identify iBALT areas in the lungs (89), and the numbers of iBALT areas are greater in mice exposed for longer periods.

Given that iBALT is associated with the most severe forms of COPD (192, 201), one could argue that iBALT contributes to pathology and is detrimental for the host. Conversely, one could argue that the elevated inflammation and lung damage in patients with more severe COPD promote iBALT formation. Consistent with this idea, patients with COPD often have elevated expression of thrombin in their airways (202, 203), which is associated with pulmonary inflammation and damage (204, 205). Interestingly, mice lacking the Serine Protease Inhibitor, SERPINE2, spontaneously develop chronic pulmonary inflammation and form iBALT-like structures in their lungs (119). Thus, pathways of damage and inflammation in chronic lung disease may promote iBALT. Finally, patients with COPD often develop bacterial infections in their lung, which exacerbates disease (206). Thus, iBALT may form as a consequence of infection and, based on studies in mice, may actually be providing a benefit to the patient, despite the severity of disease.

The development of iBALT is also associated with another chronic lung condition, hypersensitivity pneumonitis. Hypersensitivity pneumonitis is caused by a chronic exposure to environmental organic dusts or molds, resulting in immune-driven inflammation (207). Often referred to as “farmer’s lung” hypersensitivity pneumonitis is typically the result of repeated exposure to a particular pulmonary antigen, such as moldy hay. Thus, iBALT structures in the lungs of hypersensitivity pneumonitis patients are often very reactive and contain enormous germinal centers (208, 209).

Given the dramatic enlargement of iBALT areas in an antigen-driven disease like hypersensitivity pneumonitis, one might expect that patients with allergen-driven asthma would also develop extensive areas of iBALT. In fact, the appearance of iBALT-like structures (isolated aggregations of lymphoid cells or IALC) is observed in asthmatic patients and is greater in number and size compared to those in non-asthmatics (210). In addition, the appearance of iBALT-like areas in asthmatics correlates with airway wall thickening and increases in eosinophil infiltration. Furthermore, the progressive organization of iBALT positively correlates with the severity of asthma symptoms, suggesting that iBALT may be responding to external antigens and exacerbating pulmonary pathology. Similarly, patients with allergic bronchopulmonary aspergillosis also develop iBALT areas, some of which have allergen-specific IgE-expressing B cells in the germinal centers (211), again suggesting an involvement of iBALT in pathology. These observations can be mimicked in mice by sensitization and pulmonary challenge with OVA, which promotes the differentiation of OVA-specific, IgE-secreting plasma cells in iBALT structures in the lungs and increases airway hyperresponsiveness (90). However, the presence of iBALT does not always correlate with the development or progression of allergy or asthma, as a study of cross-country skiers finds iBALT at a similar frequency in normal and asthmatic individuals (independent of smoking status) and does not correlate iBALT with either respiratory allergy or airway hyperresponsiveness (212). Thus, the causal relationship between iBALT and pulmonary allergies or asthma remains enigmatic.

Well-developed iBALT is also commonly found in patients with pulmonary complications of rheumatoid arthritis (RA) (208). These structures are highly reactive, with polarized germinal centers that nearly fill the B cell follicles. Plasma cells secreting antibodies specific for citrullinated proteins are found surrounding the iBALT areas. Given that antibodies against citrullinated proteins are highly specific for RA (213) and are known to be pathologic (214), these data suggest that iBALT areas are contributing to autoimmune disease. Similar structures are observed in a subset of patients with pulmonary manifestations of Sjogren’s syndrome (SS) (208). SS is also an autoimmune disease that is characterized with an autoantibody production (215). Interestingly, the lungs of both RA and SS patients with pulmonary disease have extraordinary increases in the expression of the chemokines, CXCL13 and CXCL12 (216), which likely contribute to the recruitment of lymphocytes and the formation of iBALT areas in the lung, comparable to what is observed in the salivary glands of SS patients (217). Again, these data suggest that iBALT contributes to the local production of autoantibodies and correlates with local pathology.

Immune responses against transplanted organs are similar to autoimmune responses in that alloantigens, like autoantigens, persist forever – sometimes promoting the development of ectopic lymphoid tissues like iBALT (218). For instance, iBALT is observed around small airways in a rat model of orthotopic lung transplantation (219) and, given the local immune reactivity, is thought to contribute to the rejection of the transplanted lungs. Similarly transplanted hearts and kidneys also develop ectopic lymphoid tissues that are associated with the production of antibodies directed against donor MHC-I molecules (220). Interestingly, treatment of heart allograft recipients with LTβR–Ig fusion protein abolished the formation of tertiary lymphoid tissues, attenuated the autoantibody response, and prevented graft rejection (221), suggesting again that local lymphoid tissues play a role in local immune reactivity. However, the long-term acceptance of lung allografts is also associated with the formation of iBALT (222). In this case, the acceptance of the graft is dependent on the accumulation of Foxp3^+^ Tregs that accumulate in iBALT areas. Thus, the formation of iBALT can promote tolerance (222) as well as immune reactivity and understanding how it might perform these functions will be important for future studies to determine.

Local immune reactivity and the formation of ectopic lymphoid tissues are also important for immunity against tumors (223). For example, some patients with non-small-cell lung cancer (NSCLC) develop lymphocyte clusters, called tumor-induced BALT (Ti-BALT) (224), which are associated with more favorable clinical outcomes. Presumably, DCs within Ti-BALT present tumor-associated antigens to T cells and enhance the efficiency of the immune response. In addition, ILC3 that express natural cytotoxicity receptors (NCRs) accumulate in Ti-BALT, and their frequency positively correlates with Ti-BALT formation and negatively correlates with tumor growth (225). Thus, in this context, the presence of iBALT is associated with productive immunity rather than tolerance.

## How Does BALT Do it?

There is little doubt that iBALT promotes productive immunity to a wide variety of infectious agents. It also correlates with inflammatory lung diseases, immunity against lung tumors, and transplant rejection. These data might suggest that the presence of iBALT leads to bigger and faster immune responses, which would be “good” for immunity against infection and “bad” for autoimmunity and chronic inflammation. However, there are clear instances in which iBALT correlates with tolerance against allografts and may even reduce inflammation associated with inflammatory diseases like asthma. Thus, the function(s) of iBALT are much more complex than initially thought.

How might iBALT accelerate immune responses and simultaneously suppress inflammatory responses? One possible mechanism involves the formation of additional lymphatic vessels around the iBALT follicles (64, 208), which by efficiently gathering pulmonary DCs, might accelerate immune responses and concentrate the local inflammatory response in the areas of iBALT – away from the remainder of the lung parenchyma. One can envision this process as sequestering antigens, pathogens, and cells in iBALT areas in order to control inflammation and pathology and also to efficiently eliminate or contain pathogens, like MTB. In fact, static imaging shows that inhaled antigens and particulates, such as diesel exhaust (88) or silica (226) accumulate in iBALT areas, effectively sequestering them and potentially reducing their ability to trigger inflammation.

A similar mechanism may be acting in CCR7^−/−^ mice, which spontaneously develop iBALT in the context of rheumatoid lung disease (127, 195), but are simultaneously protected from developing bleomycin-induced pulmonary fibrosis (227). In addition, mice that develop iBALT as a consequence of autoimmunity are also protected from bleomycin-induced fibrosis (228). One possible explanation for these results is that following bleomycin administration, the iBALT areas rapidly sequester the drug or efficiently drain it out of the lung *via* lymphatics, thereby reducing its ability to trigger a fibrotic response.

One can extend this idea to antigens and allergens that are taken up by phagocytic cells in the lung. The areas of iBALT may efficiently collect antigen-bearing DCs or macrophages *via* lymphatics or other mechanisms, promoting their concentration in areas devoted to T and B cell priming, and simultaneously depleting them from the rest of the lung. In fact, plasmacytoid DCs (pDCs) in patients with asthma and in patients with mild moderate COPD are found concentrated in iBALT areas of the lung (229) where they may promote the local differentiation of Tregs (230, 231). In addition, lymphatic vessels in iBALT provide a survival niche for memory CD4 T cells by providing IL-7. Interestingly, lymphatic endothelial cells also produce cytokines like IL-33 as well as chemokines like CCL21 (68), all of which may contribute to trafficking, activation, and survival of lymphocytes. These possibilities highlight the potential regulatory function of iBALT in the context of inflammatory diseases.

## Concluding Remarks

The delicate mucosal surface of the lung is constantly exposed to pathogens and environmental antigens, but in most cases manages to generate immune responses that are sufficient to clear pathogens without causing undue damage. The presence of iBALT clearly plays a role in this process by modulating local immune responses in a way that accelerates immunity to pathogens and, in some cases, ameliorating chronic inflammation. One might argue that iBALT achieves both these effects by sequestering antigens and cells in small areas of lymphoid tissue in the lung. However, the factors that control the activity of iBALT are unclear and will undoubtedly be the focus of future studies. Once we understand the pathways that control the development and function of iBALT, we may be able to target therapies that promote or inhibit these activities, depending on the context.

## Author Contributions

All authors listed, have made substantial, direct and intellectual contribution to the work, and approved it for publication.

## Conflict of Interest Statement

The authors declare that the research was conducted in the absence of any commercial or financial relationships that could be construed as a potential conflict of interest.

## References

[1] Goodnow CC Chance encounters and organized rendezvous. Immunol Rev (1997) 156:5–10. 10.1111/j.1600-065X.1997.tb00954.x 9176695

[2] MacLennan IC Gulbranson-Judge A Toellner KM Casamayor-Palleja M Chan E Sze DM The changing preference of T and B cells for partners as T-dependent antibody responses develop. Immunol Rev (1997) 156:53–66. 10.1111/j.1600-065X.1997.tb00958.x 9176699

[3] Danilova N. The evolution of adaptive immunity. Adv Exp Med Biol (2012) 738:218–35. 10.1007/978-1-4614-1680-7_13 22399382

[4] Girard JP Moussion C Forster R. HEVs, lymphatics and homeostatic immune cell trafficking in lymph nodes. Nat Rev Immunol (2012) 12:762–73. 10.1038/nri3298 23018291

[5] Boehm T Hess I Swann JB. Evolution of lymphoid tissues. Trends Immunol (2012) 33:315–21. 10.1016/j.it.2012.02.005 22483556

[6] Amemiya CT Saha NR Zapata A. Evolution and development of immunological structures in the lamprey. Curr Opin Immunol (2007) 19:535–41. 10.1016/j.coi.2007.08.003 17875388 PMC2093943

[7] Rast JP Buckley KM Lamprey immunity is far from primitive. Proc Natl Acad Sci U S A (2013) 110:5746–7. 10.1073/pnas.1303541110 23553834 PMC3625256

[8] Rennert PD Browning JL Mebius R Mackay F Hochman PS. Surface lymphotoxin alpha/beta complex is required for the development of peripheral lymphoid organs. J Exp Med (1996) 184:1999–2006. 10.1084/jem.184.5.1999 8920886 PMC2192901

[9] Randall TD Mebius RE. The development and function of mucosal lymphoid tissues: a balancing act with micro-organisms. Mucosal Immunol (2014) 7:455–66. 10.1038/mi.2014.11 24569801

[10] Hashi H Yoshida H Honda K Fraser S Kubo H Awane M Compartmentalization of Peyer’s patch anlagen before lymphocyte entry. J Immunol (2001) 166:3702–9. 10.4049/jimmunol.166.6.3702 11238610

[11] Nishikawa S Nishikawa S Honda K Hashi H Yoshida H. Peyer’s patch organogenesis as a programmed inflammation: a hypothetical model. Cytokine Growth Factor Rev (1998) 9:213–20. 10.1016/S1359-6101(98)00014-8 9918121

[12] Krege J Seth S Hardtke S Davalos-Misslitz AC Forster R. Antigen-dependent rescue of nose-associated lymphoid tissue (NALT) development independent of LTbetaR and CXCR5 signaling. Eur J Immunol (2009) 39:2765–78. 10.1002/eji.200939422 19757439

[13] Pospisil R Mage RG. Rabbit appendix: a site of development and selection of the B cell repertoire. Curr Top Microbiol Immunol (1998) 229:59–70. 9479848 10.1007/978-3-642-71984-4_6

[14] Kendall PL Yu G Woodward EJ Thomas JW. Tertiary lymphoid structures in the pancreas promote selection of B lymphocytes in autoimmune diabetes. J Immunol (2007) 178:5643–51. 10.4049/jimmunol.178.9.5643 17442947

[15] Marinkovic T Garin A Yokota Y Fu YX Ruddle NH Furtado GC Interaction of mature CD3+CD4+ T cells with dendritic cells triggers the development of tertiary lymphoid structures in the thyroid. J Clin Invest (2006) 116:2622–32. 10.1172/JCI28993 16998590 PMC1570377

[16] Weiss JM Cufi P Le Panse R Berrih-Aknin S. The thymus in autoimmune myasthenia gravis: paradigm for a tertiary lymphoid organ. Rev Neurol (2013) 169:640–9. 10.1016/j.neurol.2013.02.005 24008049

[17] Astorri E Scrivo R Bombardieri M Picarelli G Pecorella I Porzia A CX3CL1 and CX3CR1 expression in tertiary lymphoid structures in salivary gland infiltrates: fractalkine contribution to lymphoid neogenesis in Sjogren’s syndrome. Rheumatology (2014) 53:611–20. 10.1093/rheumatology/ket401 24324211

[18] Barone F Nayar S Campos J Cloake T Withers DR Toellner KM IL-22 regulates lymphoid chemokine production and assembly of tertiary lymphoid organs. Proc Natl Acad Sci U S A (2015) 112:11024–9. 10.1073/pnas.1503315112 26286991 PMC4568258

[19] Peters A Pitcher LA Sullivan JM Mitsdoerffer M Acton SE Franz B Th17 cells induce ectopic lymphoid follicles in central nervous system tissue inflammation. Immunity (2011) 35:986–96. 10.1016/j.immuni.2011.10.015 22177922 PMC3422678

[20] Shomer NH Fox JG Juedes AE Ruddle NH. Helicobacter-induced chronic active lymphoid aggregates have characteristics of tertiary lymphoid tissue. Infect Immun (2003) 71:3572–7. 10.1128/IAI.71.6.3572-3577.2003 12761142 PMC155770

[21] Pei G Zeng R Han M Liao P Zhou X Li Y Renal interstitial infiltration and tertiary lymphoid organ neogenesis in IgA nephropathy. Clin J Am Soc Nephrol (2014) 9:255–64. 10.2215/CJN.01150113 24262509 PMC3913227

[22] Ruddle NH. Lymphatic vessels and tertiary lymphoid organs. J Clin Invest (2014) 124:953–9. 10.1172/JCI71611 24590281 PMC3934190

[23] Moyron-Quiroz JE Rangel-Moreno J Kusser K Hartson L Sprague F Goodrich S Role of inducible bronchus associated lymphoid tissue (iBALT) in respiratory immunity. Nat Med (2004) 10:927–34. 10.1038/nm1091 15311275

[24] Randall TD. Bronchus-associated lymphoid tissue (BALT) structure and function. Adv Immunol (2010) 107:187–241. 10.1016/B978-0-12-381300-8.00007-1 21034975 PMC7150010

[25] Delventhal S Hensel A Petzoldt K Pabst R. Effects of microbial stimulation on the number, size and activity of bronchus-associated lymphoid tissue (BALT) structures in the pig. Int J Exp Pathol (1992) 73:351–7. 1622845 PMC2002339

[26] Tschernig T Pabst R. Bronchus-associated lymphoid tissue (BALT) is not present in the normal adult lung but in different diseases. Pathobiology (2000) 68:1–8. 10.1159/000028109 10859525

[27] Gregson RL Davey MJ Prentice DE. Bronchus-associated lymphoid tissue (BALT) in the laboratory-bred and wild rat, *Rattus norvegicus*. Lab Anim (1979) 13:239–43. 10.1258/002367779780937735 162238

[28] Jericho KW. Intrapulmonary lymphoid tissue in mink infected with Aleutian disease virus. Res Vet Sci (1982) 32:206–12. 7079603

[29] Gould SJ Isaacson PG. Bronchus-associated lymphoid tissue (BALT) in human fetal and infant lung. J Pathol (1993) 169:229–34. 10.1002/path.1711690209 8445488

[30] Pabst R Binns RM. The immune system of the respiratory tract in pigs. Vet Immunol Immunopathol (1994) 43:151–6. 10.1016/0165-2427(94)90131-7 7856047

[31] Effendy AW Zamri-Saad M Maswati MA Ismail MS Jamil SM. Stimulation of the bronchus-associated lymphoid tissue of goats and its effect on in vitro colonization by *Pasteurella haemolytica*. Vet Res Commun (1998) 22:147–53. 10.1023/A:1006064703662 9618886

[32] Fagerland JA Arp LH. Structure and development of bronchus-associated lymphoid tissue in conventionally reared broiler chickens. Avian Dis (1993) 37:10–8. 10.2307/1591451 8452486

[33] Jeurissen SH Janse EM Koch G De Boer GF. Postnatal development of mucosa-associated lymphoid tissues in chickens. Cell Tissue Res (1989) 258:119–24. 10.1007/BF00223151 2805039

[34] Smialek M Tykalowski B Stenzel T Koncicki A. Local immunity of the respiratory mucosal system in chickens and turkeys. Pol J Vet Sci (2011) 14:291–7. 21721419 10.2478/v10181-011-0047-2

[35] Gregson RL Davey MJ Prentice DE. The response of rat bronchus-associated lymphoid tissue to local antigenic challenge. Br J Exp Pathol (1979) 60:471–82. 518816 PMC2041492

[36] Kawamata N Xu B Nishijima H Aoyama K Kusumoto M Takeuchi T Expression of endothelia and lymphocyte adhesion molecules in bronchus-associated lymphoid tissue (BALT) in adult human lung. Respir Res (2009) 10:97. 10.1186/1465-9921-10-97 19845971 PMC2772857

[37] Woodland DL Randall TD. Anatomical features of anti-viral immunity in the respiratory tract. Semin Immunol (2004) 16:163–70. 10.1016/j.smim.2004.02.003 15130500 PMC7128764

[38] Matsumoto M Fu YX Molina H Chaplin DD. Lymphotoxin-alpha-deficient and TNF receptor-I-deficient mice define developmental and functional characteristics of germinal centers. Immunol Rev (1997) 156:137–44. 10.1111/j.1600-065X.1997.tb00965.x 9176705

[39] Endres R Alimzhanov MB Plitz T Futterer A Kosco-Vilbois MH Nedospasov SA Mature follicular dendritic cell networks depend on expression of lymphotoxin beta receptor by radioresistant stromal cells and of lymphotoxin beta and tumor necrosis factor by B cells. J Exp Med (1999) 189:159–68. 10.1084/jem.189.1.159 9874572 PMC1887694

[40] Futterer A Mink K Luz A Kosco-Vilbois MH Pfeffer K. The lymphotoxin beta receptor controls organogenesis and affinity maturation in peripheral lymphoid tissues. Immunity (1998) 9:59–70. 10.1016/S1074-7613(00)80588-9 9697836

[41] Gonzalez M Mackay F Browning JL Kosco-Vilbois MH Noelle RJ. The sequential role of lymphotoxin and B cells in the development of splenic follicles. J Exp Med (1998) 187:997–1007. 10.1084/jem.187.7.997 9529316 PMC2212214

[42] Vinuesa CG Linterman MA Goodnow CC Randall KL. T cells and follicular dendritic cells in germinal center B-cell formation and selection. Immunol Rev (2010) 237:72–89. 10.1111/j.1600-065X.2010.00937.x 20727030

[43] Vinuesa CG Linterman MA Yu D MacLennan IC. Follicular helper T cells. Annu Rev Immunol (2016) 34:335–68. 10.1146/annurev-immunol-041015-055605 26907215

[44] Pabst R Tschernig T. Lymphocytes in the lung: an often neglected cell. Numbers, characterization and compartmentalization. Anat Embryol (Berl) (1995) 192:293–9. 10.1007/BF00710098 8554162

[45] Muller G Hopken UE Lipp M. The impact of CCR7 and CXCR5 on lymphoid organ development and systemic immunity. Immunol Rev (2003) 195:117–35. 10.1034/j.1600-065X.2003.00073.x 12969315

[46] Gunn MD Ngo VN Ansel KM Ekland EH Cyster JG Williams LT. A B-cell-homing chemokine made in lymphoid follicles activates Burkitt’s lymphoma receptor-1. Nature (1998) 391:799–803. 10.1038/35876 9486651

[47] Chai Q Onder L Scandella E Gil-Cruz C Perez-Shibayama C Cupovic J Maturation of lymph node fibroblastic reticular cells from myofibroblastic precursors is critical for antiviral immunity. Immunity (2013) 38:1013–24. 10.1016/j.immuni.2013.03.012 23623380 PMC7111182

[48] Cremasco V Woodruff MC Onder L Cupovic J Nieves-Bonilla JM Schildberg FA B cell homeostasis and follicle confines are governed by fibroblastic reticular cells. Nat Immunol (2014) 15:973–81. 10.1038/ni.2965 25151489 PMC4205585

[49] Luther SA Bidgol A Hargreaves DC Schmidt A Xu Y Paniyadi J Differing activities of homeostatic chemokines CCL19, CCL21, and CXCL12 in lymphocyte and dendritic cell recruitment and lymphoid neogenesis. J Immunol (2002) 169:424–33. 10.4049/jimmunol.169.1.424 12077273

[50] Mori S Nakano H Aritomi K Wang CR Gunn MD Kakiuchi T. Mice lacking expression of the chemokines CCL21-ser and CCL19 (plt mice) demonstrate delayed but enhanced T cell immune responses. J Exp Med (2001) 193:207–18. 10.1084/jem.193.2.207 11148224 PMC2193340

[51] Vassileva G Soto H Zlotnik A Nakano H Kakiuchi T Hedrick JA The reduced expression of 6Ckine in the plt mouse results from the deletion of one of two 6Ckine genes. J Exp Med (1999) 190:1183–8. 10.1084/jem.190.8.1183 10523616 PMC2195659

[52] Jang MH Sougawa N Tanaka T Hirata T Hiroi T Tohya K CCR7 is critically important for migration of dendritic cells in intestinal lamina propria to mesenteric lymph nodes. J Immunol (2006) 176:803–10. 10.4049/jimmunol.176.2.803 16393963

[53] Forster R Schubel A Breitfeld D Kremmer E Renner-Muller I Wolf E CCR7 coordinates the primary immune response by establishing functional microenvironments in secondary lymphoid organs. Cell (1999) 99:23–33. 10.1016/S0092-8674(00)80059-8 10520991

[54] Onder L Narang P Scandella E Chai Q Iolyeva M Hoorweg K IL-7-producing stromal cells are critical for lymph node remodeling. Blood (2012) 120:4675–83. 10.1182/blood-2012-03-416859 22955921 PMC3952724

[55] Link A Vogt TK Favre S Britschgi MR Acha-Orbea H Hinz B Fibroblastic reticular cells in lymph nodes regulate the homeostasis of naive T cells. Nat Immunol (2007) 8:1255–65. 10.1038/ni1513 17893676

[56] Rangel-Moreno J Moyron-Quiroz JE Hartson L Kusser K Randall TD. Pulmonary expression of CXC chemokine ligand 13, CC chemokine ligand 19, and CC chemokine ligand 21 is essential for local immunity to influenza. Proc Natl Acad Sci U S A (2007) 104:10577–82. 10.1073/pnas.0700591104 17563386 PMC1965555

[57] Miyasaka M Tanaka T Lymphocyte trafficking across high endothelial venules: dogmas and enigmas. Nat Rev Immunol (2004) 4:360–70. 10.1038/nri1354 15122201

[58] Gorfu G Rivera-Nieves J Ley K. Role of beta7 integrins in intestinal lymphocyte homing and retention. Curr Mol Med (2009) 9:836–50. 10.2174/156652409789105525 19860663 PMC2770881

[59] Sminia T van der Brugge-Gamelkoorn GJ Jeurissen SH. Structure and function of bronchus-associated lymphoid tissue (BALT). Crit Rev Immunol (1989) 9:119–50. 2663024

[60] Tango M Suzuki E Gejyo F Ushiki T. The presence of specialized epithelial cells on the bronchus-associated lymphoid tissue (BALT) in the mouse. Arch Histol Cytol (2000) 63:81–9. 10.1679/aohc.63.81 10770591

[61] Mowat AM Viney JL. The anatomical basis of intestinal immunity. Immunol Rev (1997) 156:145–66. 10.1111/j.1600-065X.1997.tb00966.x 9176706

[62] van der Brugge-Gamelkoorn GJ van de Ende M Sminia T. Changes occurring in the epithelium covering the bronchus-associated lymphoid tissue of rats after intratracheal challenge with horseradish peroxidase. Cell Tissue Res (1986) 245:439–44. 10.1007/BF00213952 3527428

[63] Kretschmer S Dethlefsen I Hagner-Benes S Marsh LM Garn H Konig P. Visualization of intrapulmonary lymph vessels in healthy and inflamed murine lung using CD90/Thy-1 as a marker. PLoS One (2013) 8:e55201. 10.1371/journal.pone.0055201 23408960 PMC3568125

[64] Baluk P Adams A Phillips K Feng J Hong YK Brown MB Preferential lymphatic growth in bronchus-associated lymphoid tissue in sustained lung inflammation. Am J Pathol (2014) 184:1577–92. 10.1016/j.ajpath.2014.01.021 24631179 PMC4005985

[65] Halle S Dujardin HC Bakocevic N Fleige H Danzer H Willenzon S Induced bronchus-associated lymphoid tissue serves as a general priming site for T cells and is maintained by dendritic cells. J Exp Med (2009) 206:2593–601. 10.1084/jem.20091472 19917776 PMC2806625

[66] Picker LJ. Mechanisms of lymphocyte homing. Curr Opin Immunol (1992) 4:277–86. 10.1016/0952-7915(92)90077-R 1418706

[67] Richert LE Harmsen AL Rynda-Apple A Wiley JA Servid AE Douglas T Inducible bronchus-associated lymphoid tissue (iBALT) synergizes with local lymph nodes during antiviral CD4+ T cell responses. Lymphat Res Biol (2013) 11:196–202. 10.1089/lrb.2013.0015 24364842 PMC3875184

[68] Shinoda K Hirahara K Iinuma T Ichikawa T Suzuki AS Sugaya K Thy1+IL-7+ lymphatic endothelial cells in iBALT provide a survival niche for memory T-helper cells in allergic airway inflammation. Proc Natl Acad Sci U S A (2016) 113:E2842–51. 10.1073/pnas.1512600113 27140620 PMC4878506

[69] Randall TD Carragher DM Rangel-Moreno J. Development of secondary lymphoid organs. Annu Rev Immunol (2008) 26:627–50. 10.1146/annurev.immunol.26.021607.090257 18370924 PMC2590644

[70] Delventhal S Brandis A Ostertag H Pabst R. Low incidence of bronchus-associated lymphoid tissue (BALT) in chronically inflamed human lungs. Virchows Arch B Cell Pathol Incl Mol Pathol (1992) 62:271–4. 10.1007/BF02899692 1359700

[71] Hiller AS Tschernig T Kleemann WJ Pabst R. Bronchus-associated lymphoid tissue (BALT) and larynx-associated lymphoid tissue (LALT) are found at different frequencies in children, adolescents and adults. Scand J Immunol (1998) 47:159–62. 10.1046/j.1365-3083.1998.00276.x 9496692

[72] Pabst R Gehrke I. Is the bronchus-associated lymphoid tissue (BALT) an integral structure of the lung in normal mammals, including humans? Am J Respir Cell Mol Biol (1990) 3:131–5. 10.1165/ajrcmb/3.2.131 2378747

[73] Tschernig T Kleemann WJ Pabst R. Bronchus-associated lymphoid tissue (BALT) in the lungs of children who had died from sudden infant death syndrome and other causes. Thorax (1995) 50:658–60. 10.1136/thx.50.6.658 7638809 PMC1021267

[74] Bienenstock J Bronchus-associated lymphoid tissue. Int Arch Allergy Appl Immunol (1985) 76(Suppl 1):62–9. 10.1159/000233736 3872267

[75] Bienenstock J Johnston N Perey DY Bronchial lymphoid tissue. I. Morphologic characteristics. Lab Invest (1973) 28:686–92. 4123478

[76] Simecka JW Davis JK Cassell GH. Distribution of Ia antigens and T lymphocyte subpopulations in rat lungs. Immunology (1986) 57:93–8. 2935490 PMC1453891

[77] Gregson RL Davey MJ Prentice DE. Postnatal development of bronchus-associated lymphoid tissue (BALT) in the rat, *Rattus norvegicus*. Lab Anim (1979) 13:231–8. 10.1258/002367779780937870 162237

[78] Rangel-Moreno J Carragher DM de la Luz Garcia-Hernandez M Hwang JY Kusser K Hartson L The development of inducible bronchus-associated lymphoid tissue depends on IL-17. Nat Immunol (2011) 12:639–46. 10.1038/ni.2053 21666689 PMC3520063

[79] Barman NN Bhattacharyya R Upadhyaya TN Baishya G. Development of bronchus-associated lymphoid tissue in goats. Lung (1996) 174:127–31. 10.1007/BF00177706 8919435

[80] Holt PG Development of bronchus associated lymphoid tissue (BALT) in human lung disease: a normal host defence mechanism awaiting therapeutic exploitation? Thorax (1993) 48:1097–8. 10.1136/thx.48.11.1097 8296250 PMC464883

[81] Jericho KW Derbyshire JB Jones JE Intrapulmonary lymphoid tissue of pigs exposed to aerosols of haemolytic streptococcus group L and porcine adenovirus. J Comp Pathol (1971) 81:1–11. 10.1016/0021-9975(71)90049-1 4326294

[82] Foo SY Zhang V Lalwani A Lynch JP Zhuang A Lam CE Regulatory T cells prevent inducible BALT formation by dampening neutrophilic inflammation. J Immunol (2015) 194:4567–76. 10.4049/jimmunol.1400909 25810394

[83] Ersch J Tschernig T Stallmach T. Frequency and potential cause of bronchus-associated lymphoid tissue in fetal lungs. Pediatr Allergy Immunol (2005) 16:295–8. 10.1111/j.1399-3038.2005.00269.x 15943591

[84] Cupedo T Jansen W Kraal G Mebius RE. Induction of secondary and tertiary lymphoid structures in the skin. Immunity (2004) 21:655–67. 10.1016/j.immuni.2004.09.006 15539152

[85] Stahl FR Heller K Halle S Keyser KA Busche A Marquardt A Nodular inflammatory foci are sites of T cell priming and control of murine cytomegalovirus infection in the neonatal lung. PLoS Pathog (2013) 9:e1003828. 10.1371/journal.ppat.1003828 24348257 PMC3861546

[86] Stahl FR Keyser KA Heller K Bischoff Y Halle S Wagner K Mck2-dependent infection of alveolar macrophages promotes replication of MCMV in nodular inflammatory foci of the neonatal lung. Mucosal Immunol (2015) 8:57–67. 10.1038/mi.2014.42 24894498

[87] Kahnert A Hopken UE Stein M Bandermann S Lipp M Kaufmann SH. *Mycobacterium tuberculosis* triggers formation of lymphoid structure in murine lungs. J Infect Dis (2007) 195:46–54. 10.1086/508894 17152008

[88] Hiramatsu K Azuma A Kudoh S Desaki M Takizawa H Sugawara I. Inhalation of diesel exhaust for three months affects major cytokine expression and induces bronchus-associated lymphoid tissue formation in murine lungs. Exp Lung Res (2003) 29:607–22. 10.1080/01902140390240140 14594659

[89] van der Strate BW Postma DS Brandsma CA Melgert BN Luinge MA Geerlings M Cigarette smoke-induced emphysema: a role for the B cell? Am J Respir Crit Care Med (2006) 173:751–8. 10.1164/rccm.200504-594OC 16399994

[90] Chvatchko Y Kosco-Vilbois MH Herren S Lefort J Bonnefoy JY. Germinal center formation and local immunoglobulin E (IgE) production in the lung after an airway antigenic challenge. J Exp Med (1996) 184:2353–60. 10.1084/jem.184.6.2353 8976189 PMC2196373

[91] Ngo VN Korner H Gunn MD Schmidt KN Riminton DS Cooper MD Lymphotoxin alpha/beta and tumor necrosis factor are required for stromal cell expression of homing chemokines in B and T cell areas of the spleen. J Exp Med (1999) 189:403–12. 10.1084/jem.189.2.403 9892622 PMC2192983

[92] Perros F Dorfmuller P Montani D Hammad H Waelput W Girerd B Pulmonary lymphoid neogenesis in idiopathic pulmonary arterial hypertension. Am J Respir Crit Care Med (2012) 185:311–21. 10.1164/rccm.201105-0927OC 22108206

[93] Hautefort A Girerd B Montani D Cohen-Kaminsky S Price L Lambrecht BN T-helper 17 cell polarization in pulmonary arterial hypertension. Chest (2015) 147:1610–20. 10.1378/chest.14-1678 25429518

[94] Fleige H Ravens S Moschovakis GL Bolter J Willenzon S Sutter G IL-17-induced CXCL12 recruits B cells and induces follicle formation in BALT in the absence of differentiated FDCs. J Exp Med (2014) 211:643–51. 10.1084/jem.20131737 24663215 PMC3978277

[95] Fletcher HA Pathan AA Berthoud TK Dunachie SJ Whelan KT Alder NC Boosting BCG vaccination with MVA85A down-regulates the immunoregulatory cytokine TGF-beta1. Vaccine (2008) 26:5269–75. 10.1016/j.vaccine.2008.07.040 18682270 PMC2631167

[96] Price PJ Banki Z Scheideler A Stoiber H Verschoor A Sutter G Complement component C5 recruits neutrophils in the absence of C3 during respiratory infection with modified vaccinia virus Ankara. J Immunol (2015) 194:1164–8. 10.4049/jimmunol.1301410 25548218

[97] Grogan JL Ouyang W. A role for Th17 cells in the regulation of tertiary lymphoid follicles. Eur J Immunol (2012) 42:2255–62. 10.1002/eji.201242656 22949324

[98] Khader SA Guglani L Rangel-Moreno J Gopal R Junecko BA Fountain JJ IL-23 is required for long-term control of *Mycobacterium tuberculosis* and B cell follicle formation in the infected lung. J Immunol (2011) 187:5402–7. 10.4049/jimmunol.1101377 22003199 PMC3208087

[99] Eidenschenk C Rutz S Liesenfeld O Ouyang W. Role of IL-22 in microbial host defense. Curr Top Microbiol Immunol (2014) 380:213–36. 10.1007/978-3-662-43492-5_10 25004820

[100] Gopal R Rangel-Moreno J Slight S Lin Y Nawar HF Fallert Junecko BA Interleukin-17-dependent CXCL13 mediates mucosal vaccine-induced immunity against tuberculosis. Mucosal Immunol (2013) 6:972–84. 10.1038/mi.2012.135 23299616 PMC3732523

[101] Corneth OB Reijmers RM Mus AM Asmawidjaja PS van Hamburg JP Papazian N Loss of IL-22 inhibits autoantibody formation in collagen-induced arthritis in mice. Eur J Immunol (2016) 46(6):1404–14. 10.1002/eji.201546241 27067635

[102] Eberl G Littman DR. The role of the nuclear hormone receptor RORgammat in the development of lymph nodes and Peyer’s patches. Immunol Rev (2003) 195:81–90. 10.1034/j.1600-065X.2003.00074.x 12969312

[103] Cupedo T Kraal G Mebius RE. The role of CD45+CD4+CD3- cells in lymphoid organ development. Immunol Rev (2002) 189:41–50. 10.1034/j.1600-065X.2002.18905.x 12445264

[104] van de Pavert SA Olivier BJ Goverse G Vondenhoff MF Greuter M Beke P Chemokine CXCL13 is essential for lymph node initiation and is induced by retinoic acid and neuronal stimulation. Nat Immunol (2009) 10:1193–9. 10.1038/ni.1789 19783990 PMC2771164

[105] Matsumoto M Mariathasan S Nahm MH Baranyay F Peschon JJ Chaplin DD. Role of lymphotoxin and the type I TNF receptor in the formation of germinal centers. Science (1996) 271:1289–91. 10.1126/science.271.5253.1289 8638112

[106] Ngo VN Cornall RJ Cyster JG. Splenic T zone development is B cell dependent. J Exp Med (2001) 194:1649–60. 10.1084/jem.194.11.1649 11733579 PMC2193532

[107] Dubey LK Lebon L Mosconi I Yang CY Scandella E Ludewig B Lymphotoxin-dependent B cell-FRC crosstalk promotes De Novo Follicle formation and antibody production following intestinal helminth infection. Cell Rep (2016) 15:1527–41. 10.1016/j.celrep.2016.04.023 27160906

[108] Fletcher AL Acton SE Knoblich K. Lymph node fibroblastic reticular cells in health and disease. Nat Rev Immunol (2015) 15:350–61. 10.1038/nri3846 25998961 PMC5152733

[109] Lu TT Browning JL. Role of the lymphotoxin/LIGHT system in the development and maintenance of reticular networks and vasculature in lymphoid tissues. Front Immunol (2014) 5:47. 10.3389/fimmu.2014.00047 24575096 PMC3920476

[110] Nagatake T Fukuyama S Kim DY Goda K Igarashi O Sato S Id2-, RORgammat-, and LTbetaR-independent initiation of lymphoid organogenesis in ocular immunity. J Exp Med (2009) 206:2351–64. 10.1084/jem.20091436 19822644 PMC2768868

[111] Yokota Y Mansouri A Mori S Sugawara S Adachi S Nishikawa S Development of peripheral lymphoid organs and natural killer cells depends on the helix-loop-helix inhibitor Id2. Nature (1999) 397:702–6. 10.1038/17812 10067894

[112] McAleer JP Kolls JK. Directing traffic: IL-17 and IL-22 coordinate pulmonary immune defense. Immunol Rev (2014) 260:129–44. 10.1111/imr.12183 24942687 PMC4066195

[113] Schwarzenberger P La Russa V Miller A Ye P Huang W Zieske A IL-17 stimulates granulopoiesis in mice: use of an alternate, novel gene therapy-derived method for in vivo evaluation of cytokines. J Immunol (1998) 161:6383–9. 9834129

[114] Manroe BL Weinberg AG Rosenfeld CR Browne R. The neonatal blood count in health and disease. I. Reference values for neutrophilic cells. J Pediatr (1979) 95:89–98. 10.1016/S0022-3476(79)80096-7 480023

[115] Weinberg AG Rosenfeld CR Manroe BL Browne R. Neonatal blood cell count in health and disease. II. Values for lymphocytes, monocytes, and eosinophils. J Pediatr (1985) 106:462–6. 10.1016/S0022-3476(85)80681-8 4038739

[116] Das A Kole L Wang L Barrios R Moorthy B Jaiswal AK. BALT development and augmentation of hyperoxic lung injury in mice deficient in NQO1 and NQO2. Free Radic Biol Med (2006) 40:1843–56. 10.1016/j.freeradbiomed.2006.01.025 16678022

[117] Bouton MC Boulaftali Y Richard B Arocas V Michel JB Jandrot-Perrus M. Emerging role of serpinE2/protease nexin-1 in hemostasis and vascular biology. Blood (2012) 119:2452–7. 10.1182/blood-2011-10-387464 22234688

[118] Baker JB Low DA Simmer RL Cunningham DD. Protease-nexin: a cellular component that links thrombin and plasminogen activator and mediates their binding to cells. Cell (1980) 21:37–45. 10.1016/0092-8674(80)90112-9 6157479

[119] Solleti SK Srisuma S Bhattacharya S Rangel-Moreno J Bijli KM Randall TD Serpine2 deficiency results in lung lymphocyte accumulation and bronchus-associated lymphoid tissue formation. FASEB J (2016). 10.1096/fj.201500159R 27059719 PMC6137455

[120] Bizios R Lai L Fenton JW II Malik AB. Thrombin-induced chemotaxis and aggregation of neutrophils. J Cell Physiol (1986) 128:485–90. 10.1002/jcp.1041280318 3745283

[121] GeurtsvanKessel CH Willart MA Bergen IM van Rijt LS Muskens F Elewaut D Dendritic cells are crucial for maintenance of tertiary lymphoid structures in the lung of influenza virus-infected mice. J Exp Med (2009) 206:2339–49. 10.1084/jem.20090410 19808255 PMC2768850

[122] Geremia A Arancibia-Carcamo CV Fleming MP Rust N Singh B Mortensen NJ IL-23-responsive innate lymphoid cells are increased in inflammatory bowel disease. J Exp Med (2011) 208:1127–33. 10.1084/jem.20101712 21576383 PMC3173242

[123] Buonocore S Ahern PP Uhlig HH Ivanov II Littman DR Maloy KJ Innate lymphoid cells drive interleukin-23-dependent innate intestinal pathology. Nature (2010) 464:1371–5. 10.1038/nature08949 20393462 PMC3796764

[124] Sutton CE Lalor SJ Sweeney CM Brereton CF Lavelle EC Mills KH. Interleukin-1 and IL-23 induce innate IL-17 production from gammadelta T cells, amplifying Th17 responses and autoimmunity. Immunity (2009) 31:331–41. 10.1016/j.immuni.2009.08.001 19682929

[125] Langrish CL Chen Y Blumenschein WM Mattson J Basham B Sedgwick JD IL-23 drives a pathogenic T cell population that induces autoimmune inflammation. J Exp Med (2005) 201:233–40. 10.1084/jem.20041257 15657292 PMC2212798

[126] Helft J Bottcher J Chakravarty P Zelenay S Huotari J Schraml BU GM-CSF mouse bone marrow cultures comprise a heterogeneous population of CD11c(+)MHCII(+) macrophages and dendritic cells. Immunity (2015) 42:1197–211. 10.1016/j.immuni.2015.05.018 26084029

[127] Kocks JR Davalos-Misslitz AC Hintzen G Ohl L Forster R. Regulatory T cells interfere with the development of bronchus-associated lymphoid tissue. J Exp Med (2007) 204:723–34. 10.1084/jem.20061424 17371929 PMC2118537

[128] Thaunat O Kerjaschki D Nicoletti A. Is defective lymphatic drainage a trigger for lymphoid neogenesis? Trends Immunol (2006) 27:441–5. 10.1016/j.it.2006.08.003 16920402

[129] Lee JJ McGarry MP Farmer SC Denzler KL Larson KA Carrigan PE Interleukin-5 expression in the lung epithelium of transgenic mice leads to pulmonary changes pathognomonic of asthma. J Exp Med (1997) 185:2143–56. 10.1084/jem.185.12.2143 9182686 PMC2196351

[130] Goya S Matsuoka H Mori M Morishita H Kida H Kobashi Y Sustained interleukin-6 signalling leads to the development of lymphoid organ-like structures in the lung. J Pathol (2003) 200:82–7. 10.1002/path.1321 12692845

[131] Botelho FM Rangel-Moreno J Fritz D Randall TD Xing Z Richards CD. Pulmonary expression of oncostatin M (OSM) promotes inducible BALT formation independently of IL-6, despite a role for IL-6 in OSM-driven pulmonary inflammation. J Immunol (2013) 191:1453–64. 10.4049/jimmunol.1203318 23797667 PMC4055037

[132] Mizoguchi C Uehara S Akira S Takatsu K. IL-5 induces IgG1 isotype switch recombination in mouse CD38-activated sIgD-positive B lymphocytes. J Immunol (1999) 162:2812–9. 10072528

[133] Kishimoto T. IL-6: from its discovery to clinical applications. Int Immunol (2010) 22:347–52. 10.1093/intimm/dxq030 20410258

[134] Ravin KA Loy M The eosinophil in infection. Clin Rev Allergy Immunol (2015) 50(2):214–27. 10.1007/s12016-015-8525-4 26690368

[135] Tecchio C Micheletti A Cassatella MA. Neutrophil-derived cytokines: facts beyond expression. Front Immunol (2014) 5:508. 10.3389/fimmu.2014.00508 25374568 PMC4204637

[136] Koga H Miyahara N Fuchimoto Y Ikeda G Waseda K Ono K Inhibition of neutrophil elastase attenuates airway hyperresponsiveness and inflammation in a mouse model of secondary allergen challenge: neutrophil elastase inhibition attenuates allergic airway responses. Respir Res (2013) 14:8. 10.1186/1465-9921-14-8 23347423 PMC3570429

[137] Meyer-Hoffert U Wiedow O. Neutrophil serine proteases: mediators of innate immune responses. Curr Opin Hematol (2011) 18:19–24. 10.1097/MOH.0b013e32834115d1 21042214

[138] Felton JM Lucas CD Rossi AG Dransfield I. Eosinophils in the lung – modulating apoptosis and efferocytosis in airway inflammation. Front Immunol (2014) 5:302. 10.3389/fimmu.2014.00302 25071763 PMC4076794

[139] Barak Y Blachar Y Levin S. Neonatal neutrophilia: possible role of a humoral granulopoietic factor. Pediatr Res (1980) 14:1026–8. 10.1203/00006450-198009000-00002 6969879

[140] Hayes SM Sirr A Jacob S Sim GK Augustin A. Role of IL-7 in the shaping of the pulmonary gamma delta T cell repertoire. J Immunol (1996) 156:2723–9. 8609389

[141] McGrath-Morrow SA Lee S Gibbs K Lopez A Collaco JM Neptune E Immune response to intrapharyngeal LPS in neonatal and juvenile mice. Am J Respir Cell Mol Biol (2015) 52:323–31. 10.1165/rcmb.2014-0100OC 25068533 PMC4370259

[142] Miethke AG Saxena V Shivakumar P Sabla GE Simmons J Chougnet CA. Post-natal paucity of regulatory T cells and control of NK cell activation in experimental biliary atresia. J Hepatol (2010) 52:718–26. 10.1016/j.jhep.2009.12.027 20347178 PMC2864543

[143] Torow N Yu K Hassani K Freitag J Schulz O Basic M Active suppression of intestinal CD4(+)TCRalphabeta(+) T-lymphocyte maturation during the postnatal period. Nat Commun (2015) 6:7725 10.1038/ncomms8725 26195040 PMC4518322

[144] Liles WC Kiener PA Ledbetter JA Aruffo A Klebanoff SJ. Differential expression of Fas (CD95) and Fas ligand on normal human phagocytes: implications for the regulation of apoptosis in neutrophils. J Exp Med (1996) 184:429–40. 10.1084/jem.184.2.429 8760796 PMC2192712

[145] Borges VM Vandivier RW McPhillips KA Kench JA Morimoto K Groshong SD TNFalpha inhibits apoptotic cell clearance in the lung, exacerbating acute inflammation. Am J Physiol Lung Cell Mol Physiol (2009) 297:L586–95. 10.1152/ajplung.90569.2008 19648283 PMC2770785

[146] Remijsen Q Kuijpers TW Wirawan E Lippens S Vandenabeele P Vanden Berghe T. Dying for a cause: NETosis, mechanisms behind an antimicrobial cell death modality. Cell Death Differ (2011) 18:581–8. 10.1038/cdd.2011.1 21293492 PMC3131909

[147] Almyroudis NG Grimm MJ Davidson BA Rohm M Urban CF Segal BH. NETosis and NADPH oxidase: at the intersection of host defense, inflammation, and injury. Front Immunol (2013) 4:45. 10.3389/fimmu.2013.00045 23459634 PMC3585429

[148] Stark MA Huo Y Burcin TL Morris MA Olson TS Ley K. Phagocytosis of apoptotic neutrophils regulates granulopoiesis via IL-23 and IL-17. Immunity (2005) 22:285–94. 10.1016/j.immuni.2005.01.011 15780986

[149] Chrysanthopoulou A Mitroulis I Apostolidou E Arelaki S Mikroulis D Konstantinidis T Neutrophil extracellular traps promote differentiation and function of fibroblasts. J Pathol (2014) 233:294–307. 10.1002/path.4359 24740698

[150] Lefrancais E Roga S Gautier V Gonzalez-de-Peredo A Monsarrat B Girard JP IL-33 is processed into mature bioactive forms by neutrophil elastase and cathepsin G. Proc Natl Acad Sci U S A (2012) 109:1673–8. 10.1073/pnas.1115884109 22307629 PMC3277172

[151] Fujita J Kawaguchi M Kokubu F Ohara G Ota K Huang SK Interleukin-33 induces interleukin-17F in bronchial epithelial cells. Allergy (2012) 67:744–50. 10.1111/j.1398-9995.2012.02825.x 22540331

[152] Soerensen CM Holmskov U Aalbaek B Boye M Heegaard PM Nielsen OL. Pulmonary infections in swine induce altered porcine surfactant protein D expression and localization to dendritic cells in bronchial-associated lymphoid tissue. Immunology (2005) 115:526–35. 10.1111/j.1365-2567.2005.02189.x 16011521 PMC1782188

[153] Zhao J Lin L Fu L Han L Zhang A Neutrophils extracellular Taps play an important role in clearance of *Streptococcus suis* in vivo. Microbiol Immunol (2016) 60(4):228–33. 10.1111/1348-0421.12367 26876770

[154] Morissette MC Jobse BN Thayaparan D Nikota JK Shen P Labiris NR Persistence of pulmonary tertiary lymphoid tissues and anti-nuclear antibodies following cessation of cigarette smoke exposure. Respir Res (2014) 15:49. 10.1186/1465-9921-15-49 24754996 PMC4021094

[155] Aloisi F Pujol-Borrell R. Lymphoid neogenesis in chronic inflammatory diseases. Nat Rev Immunol (2006) 6:205–17. 10.1038/nri1786 16498451

[156] Columba-Cabezas S Griguoli M Rosicarelli B Magliozzi R Ria F Serafini B Suppression of established experimental autoimmune encephalomyelitis and formation of meningeal lymphoid follicles by lymphotoxin beta receptor-Ig fusion protein. J Neuroimmunol (2006) 179:76–86. 10.1016/j.jneuroim.2006.06.015 16870269

[157] Aloisi F Columba-Cabezas S Franciotta D Rosicarelli B Magliozzi R Reynolds R Lymphoid chemokines in chronic neuroinflammation. J Neuroimmunol (2008) 198:106–12. 10.1016/j.jneuroim.2008.04.025 18539341 PMC7125843

[158] Gaublomme JT Yosef N Lee Y Gertner RS Yang LV Wu C Single-cell genomics unveils critical regulators of Th17 cell pathogenicity. Cell (2015) 163:1400–12. 10.1016/j.cell.2015.11.009 26607794 PMC4671824

[159] Pikor NB Astarita JL Summers-Deluca L Galicia G Qu J Ward LA Integration of Th17- and lymphotoxin-derived signals initiates meningeal-resident stromal cell remodeling to propagate neuroinflammation. Immunity (2015) 43:1160–73. 10.1016/j.immuni.2015.11.010 26682987

[160] Astorri E Bombardieri M Gabba S Peakman M Pozzilli P Pitzalis C. Evolution of ectopic lymphoid neogenesis and in situ autoantibody production in autoimmune nonobese diabetic mice: cellular and molecular characterization of tertiary lymphoid structures in pancreatic islets. J Immunol (2010) 185:3359–68. 10.4049/jimmunol.1001836 20713891

[161] Henry RA Kendall PL. CXCL13 blockade disrupts B lymphocyte organization in tertiary lymphoid structures without altering B cell receptor bias or preventing diabetes in nonobese diabetic mice. J Immunol (2010) 185:1460–5. 10.4049/jimmunol.0903710 20574003 PMC3824617

[162] Luther SA Lopez T Bai W Hanahan D Cyster JG. BLC expression in pancreatic islets causes B cell recruitment and lymphotoxin-dependent lymphoid neogenesis. Immunity (2000) 12:471–81. 10.1016/S1074-7613(00)80199-5 10843380

[163] Fan L Reilly CR Luo Y Dorf ME Lo D. Cutting edge: ectopic expression of the chemokine TCA4/SLC is sufficient to trigger lymphoid neogenesis. J Immunol (2000) 164:3955–9. 10.4049/jimmunol.164.8.3955 10754285

[164] Luther SA Ansel KM Cyster JG. Overlapping roles of CXCL13, interleukin 7 receptor alpha, and CCR7 ligands in lymph node development. J Exp Med (2003) 197:1191–8. 10.1084/jem.20021294 12732660 PMC2193976

[165] Kratz A Campos-Neto A Hanson MS Ruddle NH. Chronic inflammation caused by lymphotoxin is lymphoid neogenesis. J Exp Med (1996) 183:1461–72. 10.1084/jem.183.4.1461 8666904 PMC2192477

[166] Picarella DE Kratz A Li CB Ruddle NH Flavell RA. Transgenic tumor necrosis factor (TNF)-alpha production in pancreatic islets leads to insulitis, not diabetes. Distinct patterns of inflammation in TNF-alpha and TNF-beta transgenic mice. J Immunol (1993) 150:4136–50. 7682590

[167] Chen SC Vassileva G Kinsley D Holzmann S Manfra D Wiekowski MT Ectopic expression of the murine chemokines CCL21a and CCL21b induces the formation of lymph node-like structures in pancreas, but not skin, of transgenic mice. J Immunol (2002) 168:1001–8. 10.4049/jimmunol.168.3.1001 11801632

[168] Muniz LR Pacer ME Lira SA Furtado GC. A critical role for dendritic cells in the formation of lymphatic vessels within tertiary lymphoid structures. J Immunol (2011) 187:828–34. 10.4049/jimmunol.1004233 21666055 PMC3137511

[169] Martin AP Coronel EC Sano G Chen SC Vassileva G Canasto-Chibuque C A novel model for lymphocytic infiltration of the thyroid gland generated by transgenic expression of the CC chemokine CCL21. J Immunol (2004) 173:4791–8. 10.4049/jimmunol.173.8.4791 15470018

[170] Furtado GC Marinkovic T Martin AP Garin A Hoch B Hubner W Lymphotoxin beta receptor signaling is required for inflammatory lymphangiogenesis in the thyroid. Proc Natl Acad Sci U S A (2007) 104:5026–31. 10.1073/pnas.0606697104 17360402 PMC1829258

[171] Marchesi F Martin AP Thirunarayanan N Devany E Mayer L Grisotto MG CXCL13 expression in the gut promotes accumulation of IL-22-producing lymphoid tissue-inducer cells, and formation of isolated lymphoid follicles. Mucosal Immunol (2009) 2:486–94. 10.1038/mi.2009.113 19741597

[172] Pearson C Uhlig HH Powrie F. Lymphoid microenvironments and innate lymphoid cells in the gut. Trends Immunol (2012) 33:289–96. 10.1016/j.it.2012.04.004 22578693

[173] De Togni P Goellner J Ruddle NH Streeter PR Fick A Mariathasan S Abnormal development of peripheral lymphoid organs in mice deficient in lymphotoxin. Science (1994) 264:703–7. 10.1126/science.8171322 8171322

[174] Lund FE Partida-Sanchez S Lee BO Kusser KL Hartson L Hogan RJ Lymphotoxin-alpha-deficient mice make delayed, but effective, T and B cell responses to influenza. J Immunol (2002) 169:5236–43. 10.4049/jimmunol.169.9.5236 12391242

[175] Day TA Koch M Nouailles G Jacobsen M Kosmiadi GA Miekley D Secondary lymphoid organs are dispensable for the development of T-cell-mediated immunity during tuberculosis. Eur J Immunol (2010) 40:1663–73. 10.1002/eji.201040299 20222088

[176] Constant SL Brogdon JL Piggott DA Herrick CA Visintin I Ruddle NH Resident lung antigen-presenting cells have the capacity to promote Th2 T cell differentiation in situ. J Clin Invest (2002) 110:1441–8. 10.1172/JCI0216109 12438442 PMC151814

[177] Kashino SS Vallerskog T Martens G Troudt J Keyser A Taylor J Initiation of acquired immunity in the lungs of mice lacking lymph nodes after infection with aerosolized *Mycobacterium tuberculosis*. Am J Pathol (2010) 176:198–204. 10.2353/ajpath.2010.090446 20008132 PMC2797882

[178] Kabashima K Banks TA Ansel KM Lu TT Ware CF Cyster JG. Intrinsic lymphotoxin-beta receptor requirement for homeostasis of lymphoid tissue dendritic cells. Immunity (2005) 22:439–50. 10.1016/j.immuni.2005.02.007 15845449

[179] Mebius RE. Organogenesis of lymphoid tissues. Nat Rev Immunol (2003) 3:292–303. 10.1038/nri1054 12669020

[180] Moyron-Quiroz JE Rangel-Moreno J Hartson L Kusser K Tighe MP Klonowski KD Persistence and responsiveness of immunologic memory in the absence of secondary lymphoid organs. Immunity (2006) 25:643–54. 10.1016/j.immuni.2006.08.022 17045819

[181] Wiley JA Richert LE Swain SD Harmsen A Barnard DL Randall TD Inducible bronchus-associated lymphoid tissue elicited by a protein cage nanoparticle enhances protection in mice against diverse respiratory viruses. PLoS One (2009) 4:e7142. 10.1371/journal.pone.0007142 19774076 PMC2743193

[182] Chiavolini D Rangel-Moreno J Berg G Christian K Oliveira-Nascimento L Weir S Bronchus-associated lymphoid tissue (BALT) and survival in a vaccine mouse model of tularemia. PLoS One (2010) 5:e11156. 10.1371/journal.pone.0011156 20585390 PMC2886834

[183] Ramakrishnan L. Revisiting the role of the granuloma in tuberculosis. Nat Rev Immunol (2012) 12:352–66. 10.1038/nri3211 22517424

[184] Khader SA Bell GK Pearl JE Fountain JJ Rangel-Moreno J Cilley GE IL-23 and IL-17 in the establishment of protective pulmonary CD4+ T cell responses after vaccination and during *Mycobacterium tuberculosis* challenge. Nat Immunol (2007) 8:369–77. 10.1038/ni1449 17351619

[185] Khader SA Rangel-Moreno J Fountain JJ Martino CA Reiley WW Pearl JE In a murine tuberculosis model, the absence of homeostatic chemokines delays granuloma formation and protective immunity. J Immunol (2009) 183:8004–14. 10.4049/jimmunol.0901937 19933855 PMC2799945

[186] Slight SR Rangel-Moreno J Gopal R Lin Y Fallert Junecko BA Mehra S CXCR5(+) T helper cells mediate protective immunity against tuberculosis. J Clin Invest (2013) 123:712–26. 10.1172/JCI65728 23281399 PMC3561804

[187] Ulrichs T Kosmiadi GA Trusov V Jorg S Pradl L Titukhina M Human tuberculous granulomas induce peripheral lymphoid follicle-like structures to orchestrate local host defence in the lung. J Pathol (2004) 204:217–28. 10.1002/path.1628 15376257

[188] Kaushal D Foreman TW Gautam US Alvarez X Adekambi T Rangel-Moreno J Mucosal vaccination with attenuated *Mycobacterium tuberculosis* induces strong central memory responses and protects against tuberculosis. Nat Commun (2015) 6:8533. 10.1038/ncomms9533 26460802 PMC4608260

[189] Meuwissen HJ Hussain M Bronchus-associated lymphoid tissue in human lung: correlation of hyperplasia with chronic pulmonary disease. Clin Immunol Immunopathol (1982) 23:548–61. 10.1016/0090-1229(82)90139-8 7105501

[190] Litsiou E Semitekolou M Galani IE Morianos I Tsoutsa A Kara P CXCL13 production in B cells via Toll-like receptor/lymphotoxin receptor signaling is involved in lymphoid neogenesis in chronic obstructive pulmonary disease. Am J Respir Crit Care Med (2013) 187:1194–202. 10.1164/rccm.201208-1543OC 23525932

[191] Pauwels RA Rabe KF. Burden and clinical features of chronic obstructive pulmonary disease (COPD). Lancet (2004) 364:613–20. 10.1016/S0140-6736(04)16855-4 15313363

[192] Hogg JC Chu F Utokaparch S Woods R Elliott WM Buzatu L The nature of small-airway obstruction in chronic obstructive pulmonary disease. N Engl J Med (2004) 350:2645–53. 10.1056/NEJMoa032158 15215480

[193] Kelsen SG Aksoy MO Georgy M Hershman R Ji R Li X Lymphoid follicle cells in chronic obstructive pulmonary disease overexpress the chemokine receptor CXCR3. Am J Respir Crit Care Med (2009) 179:799–805. 10.1164/rccm.200807-1089OC 19218194 PMC5803653

[194] Demoor T Bracke KR Maes T Vandooren B Elewaut D Pilette C Role of lymphotoxin-alpha in cigarette smoke-induced inflammation and lymphoid neogenesis. Eur Respir J (2009) 34:405–16. 10.1183/09031936.00101408 19164352

[195] Demoor T Bracke KR Vermaelen KY Dupont L Joos GF Brusselle GG. CCR7 modulates pulmonary and lymph node inflammatory responses in cigarette smoke-exposed mice. J Immunol (2009) 183:8186–94. 10.4049/jimmunol.0902015 19923454

[196] Polverino F Cosio BG Pons J Laucho-Contreras M Tejera P Iglesias A B cell-activating factor. An orchestrator of lymphoid follicles in severe chronic obstructive pulmonary disease. Am J Respir Crit Care Med (2015) 192:695–705. 10.1164/rccm.201501-0107OC 26073875 PMC4595676

[197] John-Schuster G Gunter S Hager K Conlon TM Eickelberg O Yildirim AO. Inflammaging increases susceptibility to cigarette smoke-induced COPD. Oncotarget (2015) 7:30068–30083. 10.18632/oncotarget.4027 26284585 PMC5058664

[198] Mori M Andersson CK Svedberg KA Glader P Bergqvist A Shikhagaie M Appearance of remodelled and dendritic cell-rich alveolar-lymphoid interfaces provides a structural basis for increased alveolar antigen uptake in chronic obstructive pulmonary disease. Thorax (2013) 68:521–31. 10.1136/thoraxjnl-2012-202879 23412435

[199] Van Pottelberge GR Bracke KR Demedts IK De Rijck K Reinartz SM van Drunen CM Selective accumulation of langerhans-type dendritic cells in small airways of patients with COPD. Respir Res (2010) 11:35. 10.1186/1465-9921-11-35 20307269 PMC2858735

[200] Demedts IK Bracke KR Van Pottelberge G Testelmans D Verleden GM Vermassen FE Accumulation of dendritic cells and increased CCL20 levels in the airways of patients with chronic obstructive pulmonary disease. Am J Respir Crit Care Med (2007) 175:998–1005. 10.1164/rccm.200608-1113OC 17332482

[201] Gosman MM Willemse BW Jansen DF Lapperre TS van Schadewijk A Hiemstra PS Increased number of B-cells in bronchial biopsies in COPD. Eur Respir J (2006) 27:60–4. 10.1183/09031936.06.00007005 16387936

[202] Jankowski M Undas A Kaczmarek P Butenas S. Activated factor XI and tissue factor in chronic obstructive pulmonary disease: links with inflammation and thrombin generation. Thromb Res (2011) 127:242–6. 10.1016/j.thromres.2010.11.005 21236471 PMC3042502

[203] Ashitani J Mukae H Arimura Y Matsukura S. Elevated plasma procoagulant and fibrinolytic markers in patients with chronic obstructive pulmonary disease. Intern Med (2002) 41:181–5. 10.2169/internalmedicine.41.181 11929177

[204] Leonard A Marando C Rahman A Fazal F Thrombin selectively engages LIM kinase 1 and slingshot-1L phosphatase to regulate NF-kappaB activation and endothelial cell inflammation. Am J Physiol Lung Cell Mol Physiol (2013) 305:L651–64. 10.1152/ajplung.00071.2013 24039253 PMC3840277

[205] Lin CH Yu MC Chiang CC Bien MY Chien MH Chen BC Thrombin-induced NF-kappaB activation and IL-8/CXCL8 release is mediated by c-Src-dependent Shc, Raf-1, and ERK pathways in lung epithelial cells. Cell Signal (2013) 25:1166–75. 10.1016/j.cellsig.2013.01.018 23357535

[206] Sethi S. New developments in the pathogenesis of acute exacerbations of chronic obstructive pulmonary disease. Curr Opin Infect Dis (2004) 17:113–9. 10.1097/00001432-200404000-00008 15021050

[207] Bourke SJ Dalphin JC Boyd G McSharry C Baldwin CI Calvert JE. Hypersensitivity pneumonitis: current concepts. Eur Respir J Suppl (2001) 32:81s–92s. 11816827

[208] Rangel-Moreno J Hartson L Navarro C Gaxiola M Selman M Randall TD. Inducible bronchus-associated lymphoid tissue (iBALT) in patients with pulmonary complications of rheumatoid arthritis. J Clin Invest (2006) 116:3183–94. 10.1172/JCI28756 17143328 PMC1678820

[209] Suda T Chida K Hayakawa H Imokawa S Iwata M Nakamura H Development of bronchus-associated lymphoid tissue in chronic hypersensitivity pneumonitis. Chest (1999) 115:357–63. 10.1378/chest.115.2.357 10027432

[210] Elliot JG Jensen CM Mutavdzic S Lamb JP Carroll NG James AL. Aggregations of lymphoid cells in the airways of nonsmokers, smokers, and subjects with asthma. Am J Respir Crit Care Med (2004) 169:712–8. 10.1164/rccm.200308-1167OC 14711796

[211] Slavin RG Gleich GJ Hutcheson PS Kephart GM Knutsen AP Tsai CC Localization of IgE to lung germinal lymphoid follicles in a patient with allergic bronchopulmonary aspergillosis. J Allergy Clin Immunol (1992) 90:1006–8. 10.1016/0091-6749(92)90479-L 1460191

[212] Sue-Chu M Karjalainen EM Altraja A Laitinen A Laitinen LA Naess AB Lymphoid aggregates in endobronchial biopsies from young elite cross-country skiers. Am J Respir Crit Care Med (1998) 158:597–601. 10.1164/ajrccm.158.2.9711012 9700140

[213] Niewold TB Harrison MJ Paget SA. Anti-CCP antibody testing as a diagnostic and prognostic tool in rheumatoid arthritis. QJM (2007) 100:193–201. 10.1093/qjmed/hcm015 17434910

[214] Schellekens GA de Jong BA van den Hoogen FH van de Putte LB van Venrooij WJ. Citrulline is an essential constituent of antigenic determinants recognized by rheumatoid arthritis-specific autoantibodies. J Clin Invest (1998) 101:273–81. 10.1172/JCI1316 9421490 PMC508564

[215] Horsfall AC Rose LM Maini RN Autoantibody synthesis in salivary glands of Sjogren’s syndrome patients. J Autoimmun (1989) 2:559–68. 10.1016/0896-8411(89)90189-3 2789658

[216] Bleul CC Fuhlbrigge RC Casasnovas JM Aiuti A Springer TA. A highly efficacious lymphocyte chemoattractant, stromal cell-derived factor 1 (SDF-1). J Exp Med (1996) 184:1101–9. 10.1084/jem.184.3.1101 9064327 PMC2192798

[217] Amft N Curnow SJ Scheel-Toellner D Devadas A Oates J Crocker J Ectopic expression of the B cell-attracting chemokine BCA-1 (CXCL13) on endothelial cells and within lymphoid follicles contributes to the establishment of germinal center-like structures in Sjogren’s syndrome. Arthritis Rheum (2001) 44:2633–41. 10.1002/1529-0131(200111)44:11<2633::AID-ART443>3.0.CO;2-9 11710719

[218] Sato M Hirayama S Hwang DM Lara-Guerra H Wagnetz D Waddell TK The role of intrapulmonary de novo lymphoid tissue in obliterative bronchiolitis after lung transplantation. J Immunol (2009) 182:7307–16. 10.4049/jimmunol.0803606 19454728

[219] van Panhuys N Perret R Prout M Ronchese F Le Gros G. Effector lymphoid tissue and its crucial role in protective immunity. Trends Immunol (2005) 26:242–7. 10.1016/j.it.2005.03.005 15866236

[220] Thaunat O Field AC Dai J Louedec L Patey N Bloch MF Lymphoid neogenesis in chronic rejection: evidence for a local humoral alloimmune response. Proc Natl Acad Sci U S A (2005) 102:14723–8. 10.1073/pnas.0507223102 16192350 PMC1253595

[221] Motallebzadeh R Rehakova S Conlon TM Win TS Callaghan CJ Goddard M Blocking lymphotoxin signaling abrogates the development of ectopic lymphoid tissue within cardiac allografts and inhibits effector antibody responses. FASEB J (2012) 26:51–62. 10.1096/fj.11-186973 21926237

[222] Li W Bribriesco AC Nava RG Brescia AA Ibricevic A Spahn JH Lung transplant acceptance is facilitated by early events in the graft and is associated with lymphoid neogenesis. Mucosal Immunol (2012) 5:544–54. 10.1038/mi.2012.30 22549742 PMC3425714

[223] Randall TD Kern JA Tertiary lymphoid structures target the antitumor immune response to lung cancer. Am J Respir Crit Care Med (2014) 189:767–9. 10.1164/rccm.201402-0317ED 24684357 PMC4225836

[224] Dieu-Nosjean MC Antoine M Danel C Heudes D Wislez M Poulot V Long-term survival for patients with non-small-cell lung cancer with intratumoral lymphoid structures. J Clin Oncol (2008) 26:4410–7. 10.1200/JCO.2007.15.0284 18802153

[225] Carrega P Loiacono F Di Carlo E Scaramuccia A Mora M Conte R NCR(+)ILC3 concentrate in human lung cancer and associate with intratumoral lymphoid structures. Nat Commun (2015) 6:8280. 10.1038/ncomms9280 26395069

[226] Davis GS Pfeiffer LM Hemenway DR. Expansion of interferon-gamma-producing lung lymphocytes in mouse silicosis. Am J Respir Cell Mol Biol (1999) 20:813–24. 10.1165/ajrcmb.20.4.3407 10101015

[227] Trujillo G Hartigan AJ Hogaboam CM. T regulatory cells and attenuated bleomycin-induced fibrosis in lungs of CCR7-/- mice. Fibrogenesis Tissue Repair (2010) 3:18. 10.1186/1755-1536-3-18 20815874 PMC2940820

[228] Shilling RA Williams JW Perera J Berry E Wu Q Cummings OW Autoreactive T and B cells induce the development of bronchus-associated lymphoid tissue in the lung. Am J Respir Cell Mol Biol (2013) 48:406–14. 10.1165/rcmb.2012-0065OC 23371062 PMC3653607

[229] Van Pottelberge GR Bracke KR Van den Broeck S Reinartz SM van Drunen CM Wouters EF Plasmacytoid dendritic cells in pulmonary lymphoid follicles of patients with COPD. Eur Respir J (2010) 36:781–91. 10.1183/09031936.00140409 20351031

[230] Gilliet M Liu YJ. Generation of human CD8 T regulatory cells by CD40 ligand-activated plasmacytoid dendritic cells. J Exp Med (2002) 195:695–704. 10.1084/jem.20011603 11901196 PMC2193733

[231] Puccetti P Fallarino F. Generation of T cell regulatory activity by plasmacytoid dendritic cells and tryptophan catabolism. Blood Cells Mol Dis (2008) 40:101–5. 10.1016/j.bcmd.2007.06.026 17881253

